# An Immunogenic and Slow-Growing Cryptococcal Strain Induces a Chronic Granulomatous Infection in Murine Lungs

**DOI:** 10.1128/iai.00580-21

**Published:** 2022-05-19

**Authors:** Calla L. Telzrow, Shannon Esher Righi, Natalia Castro-Lopez, Althea Campuzano, Jacob T. Brooks, John M. Carney, Floyd L. Wormley, J. Andrew Alspaugh

**Affiliations:** a Department of Medicine, Duke University School of Medicine, Durham, North Carolina, USA; b Department of Molecular Genetics and Microbiology, Duke University School of Medicine, Durham, North Carolina, USA; c Department of Microbiology and Immunology, Tulane University School of Medicine, New Orleans, Louisiana, USA; d Department of Biology, University of Texas at San Antonio, San Antonio, Texas, USA; e Department of Biology, Texas Christian University, Fort Worth, Texas, USA; f Department of Physics and Astronomy, University of North Carolina at Chapel Hill, Chapel Hill, North Carolina, USA; g Department of Pathology, Duke University School of Medicine, Durham, North Carolina, USA; Tulane School of Medicine

**Keywords:** *Cryptococcus neoformans*, granuloma, GM-CSF, cell wall, Titan cell, cell cycle defects, hypoxia

## Abstract

Many successful pathogens cause latent infections, remaining dormant within the host for years but retaining the ability to reactivate to cause symptomatic disease. The human opportunistic fungal pathogen Cryptococcus neoformans establishes latent pulmonary infections in immunocompetent individuals upon inhalation from the environment. These latent infections are frequently characterized by granulomas, or foci of chronic inflammation, that contain dormant and persistent cryptococcal cells. Immunosuppression can cause these granulomas to break down and release fungal cells that proliferate, disseminate, and eventually cause lethal cryptococcosis. This course of fungal latency and reactivation is understudied due to limited models, as chronic pulmonary granulomas do not typically form in mouse cryptococcal infections. A loss-of-function mutation in the Cryptococcus-specific *MAR1* gene was previously described to alter cell surface remodeling in response to host signals. Here, we demonstrate that the *mar1*Δ mutant strain persists long term in a murine inhalation model of cryptococcosis, inducing a chronic pulmonary granulomatous response. We find that murine infections with the *mar1*Δ mutant strain are characterized by reduced fungal burden, likely due to the low growth rate of the *mar1*Δ mutant strain at physiological temperature, and an altered host immune response, likely due to inability of the *mar1*Δ mutant strain to properly employ virulence factors. We propose that this combination of features in the *mar1*Δ mutant strain collectively promotes the induction of a more chronic inflammatory response and enables long-term fungal persistence within these granulomatous regions.

## INTRODUCTION

Granulomas are complex foci of chronic inflammation that form in response to many stimuli, including microbial infections. A hallmark of indolent infections such as tuberculosis disease, granulomas are often characterized by multinucleated giant cells, epithelioid macrophages, and dormant and/or slowly proliferating microorganisms (1–4). The traditional understanding of the granuloma considered it to be a host-directed defense response that restricts microbial access to nutrients and oxygen, resulting in an immune microenvironment that limits microbial proliferation and prevents dissemination (3, 4). However, more recent work has demonstrated that granulomas are a dynamic component of the complex host-microbial “arms race.” In addition to serving as a host-directed protection mechanism, microorganisms can exploit the granuloma as a microniche for long-term survival in the host, where they remain shielded from immune detection until microbial reactivation (3–5). Although most work on granulomas has been conducted in the context of mycobacterial infections, many other infectious microorganisms induce granuloma formation in the human lung (6).

The fungal pathogen Cryptococcus neoformans is a significant cause of pneumonia and fatal meningoencephalitis in immunocompromised populations, resulting in more than 180,000 deaths annually (7). Primary infection occurs upon inhalation of environmental C. neoformans cells and/or spores, often early in life (4, 8). Immunocompetent hosts typically control the primary infection, with fungi remaining dormant but viable within lung-associated granulomas (9). As a result, immunocompetent hosts rarely manifest infection-related symptoms during this stage of latency (10). However, this chronic, latent infection can reactivate when a previously exposed individual becomes immunocompromised, especially in the setting of CD4^+^ T cell functional deficiency due to HIV infection, organ transplantation, or immunosenescence (2, 4, 7, 11). Breakdown of the cryptococcal granuloma structure results in fungal proliferation and systemic dissemination, including to the central nervous system.

The reactivation of fungal cells from granulomas is an understudied facet of cryptococcal disease, largely due to limited experimental models. Although the mouse is the most well-characterized and commonly used animal model to study Cryptococcus-host interactions, most murine models do not form chronic granulomas in response to clinically relevant isolates of C. neoformans (12). As a result, most murine experiments focus on primary cryptococcal infection and subsequent systemic dissemination. To explore cryptococcal latency and reactivation, investigators have adopted models of cryptococcosis in rabbits (13) and rats (14, 15) or employed less virulent C. neoformans strains in mice (12, 16, 17). Recently, a novel latent model was reported in which pulmonary granulomas form in mice in response to infection with the *gcs1*Δ mutant strain lacking the glucosylceramide synthase (18–21). Infection with *gcs1*Δ mutant cells results in chronic granulomas in the lungs containing persistent *gcs1*Δ mutant cells that can reactivate and disseminate upon immunosuppression (22).

We recently reported the identification and characterization of the C. neoformans *MAR1* gene, which is required for cell surface remodeling in response to the host environment (23). The *mar1*Δ loss-of-function mutant strain displays altered cell surface features when exposed to host physiological conditions, including decreased cell wall glucans and mannans, increased exposure of cell wall chitin, and impaired polysaccharide capsule attachment (23). These cell surface alterations make the *mar1*Δ mutant strain more immunogenic than the wild-type (WT) strain, resulting in enhanced macrophage activation *in vitro* and hypovirulence in a murine inhalation model of cryptococcosis (23). We report here that inoculation with the *mar1*Δ mutant strain induces a pulmonary granulomatous response in mice, resulting in a chronic and persistent infection. Furthermore, we describe both host and fungal factors that contribute to this granulomatous response. From the host perspective, granulocyte-macrophage colony-stimulating factor (GM-CSF) signaling, a known contributor to granuloma formation (17, 24–26), is similarly required for the formation of granulomatous regions of infection in this model. From the fungal perspective, murine infections with the *mar1*Δ mutant strain are characterized by reduced fungal burden and an altered host immune response. Specifically, during early stages of infection, the *mar1*Δ mutant strain induces WT strain-like pulmonary cytokine and leukocyte responses, despite its reduction in fungal burden. As infection proceeds, murine lungs infected with the WT strain exhibit progressive increases in pulmonary cytokines and leukocytes, while murine lungs infected with the *mar1*Δ mutant strain display more stable levels of pulmonary cytokines and leukocytes. *In vitro* phenotypic studies demonstrate that the *mar1*Δ mutant strain has slow growth at mammalian body temperature and is impaired in virulence factor employment, specifically Titan cell formation and polysaccharide capsule extension. We propose that the low growth rate of the *mar1*Δ mutant strain at mammalian body temperature drives its reduced fungal burden throughout the course of infection. Furthermore, we propose that the inability of the *mar1*Δ mutant strain to properly employ virulence factors drives the altered pulmonary immune response observed throughout the course of infection. These observations suggest that this combination of *mar1*Δ mutant strain phenotypes is responsible for the observed granulomatous response and the ability of the *mar1*Δ mutant strain to survive and persist within these granulomatous regions long term. Because *MAR1* is a Cryptococcus-specific gene, this model represents a unique addition to the tools available to study chronic, and potentially latent, cryptococcal disease.

## RESULTS

### Inoculation with the *mar1*Δ mutant strain induces a granulomatous response in murine lungs.

**FIG 1:**
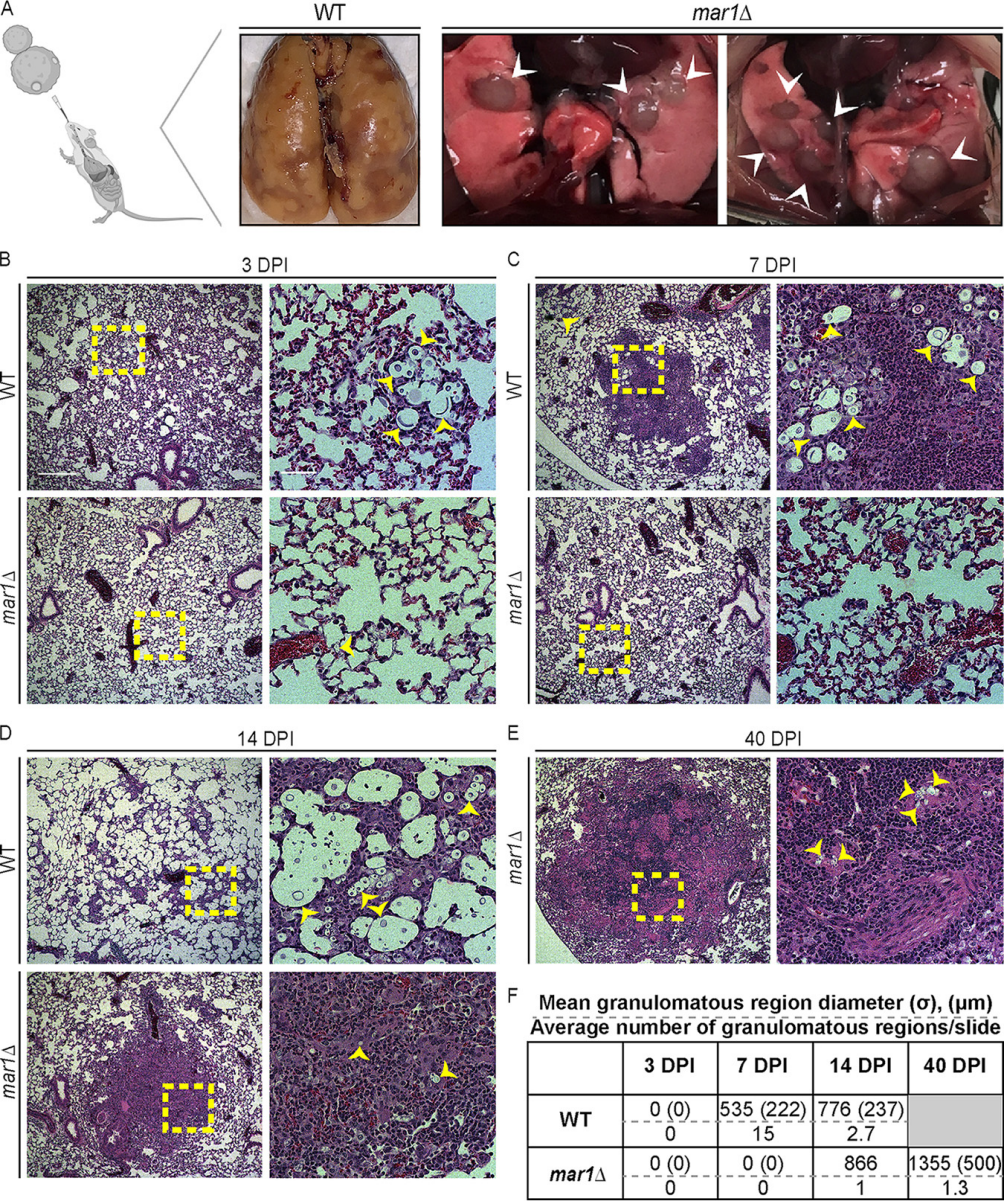
Pulmonary granulomatous region formation in murine cryptococcal infections. (A) Dissected lungs of female C57BL/6 mice infected with 1 × 10^5^ cells of the WT strain or *mar1*Δ mutant strain were harvested at clinical endpoints corresponding to imminent mortality. Gross organ examination revealed large, well-circumscribed inflammatory regions (white arrowheads) in *mar1*Δ mutant strain-inoculated lungs compared to diffuse lung injury in WT strain-inoculated lungs. (Created with BioRender.com). (B to E) Lungs of female C57BL/6 mice inoculated with 1 × 10^5^ cells of the WT strain or the *mar1*Δ mutant strain sacrificed at the predetermined endpoints of 3 (B), 7 (C), 14 (D), and 40 (E) dpi were harvested for histopathological analyses. Hematoxylin and eosin staining was utilized to visualize microscopic lung pathology (fungal cells [yellow arrowheads], inset [yellow boxes]). The 5× scale bar (left) is 250 μm, and the 10× scale bar (right) is 50 μm. (F) Granulomatous region diameter (in micrometers) was measured using Fiji. Data are summarized from one slide from three mice per strain per time point. σ, standard deviation (micrometers). The gray box indicates no experimental subjects can be assessed at this time point.

We previously observed that the *mar1*Δ mutant strain is hypovirulent compared to the wild-type (WT) strain in a murine inhalation model of cryptococcosis, despite having a more immunogenic cell surface than the WT strain (23). Highly immunogenic fungal strains often induce a hyperinflammatory response that is detrimental to the host, resulting in hypervirulence (27–29). We therefore explored in greater detail the mechanisms by which the highly immunogenic *mar1*Δ mutant strain simultaneously activates and is controlled by the host immune response. As an initial investigation into the interactions between the *mar1*Δ mutant strain and the host, we assessed the gross appearance of infected lungs from C57BL/6 mice from our previously reported murine inhalation infection experiment (23). In this experiment, C57BL/6 and BALB/c mice inoculated with the WT strain succumbed to cryptococcal infection between 10 and 30 days postinoculation (dpi) (23). In contrast, mice inoculated with the *mar1*Δ mutant strain displayed prolonged survival, with nearly 40% of the C57BL/6 mice and all the BALB/c mice surviving beyond 40 dpi (23). Gross histological analyses of C57BL/6 mice at the time of sacrifice revealed diffuse injury patterns throughout the entirety of WT strain-inoculated lungs (Fig. 1A). In contrast, *mar1*Δ mutant strain-inoculated lungs displayed large, well-circumscribed inflammatory foci surrounded by healthy-appearing lung tissue (Fig. 1A). These differences in the gross pathology suggested unique interactions between the *mar1*Δ mutant strain and the murine lung.

To examine histopathological features of infected murine lungs at specific time points throughout the course of infection, we replicated the experimental approach used in Fig. 1A, inoculating C57BL/6 mice by inhalation with the WT strain or the *mar1*Δ mutant strain and harvesting infected lungs at predetermined time points. At 3 dpi, an early time point in infection during which all inoculated mice still appeared healthy, both WT strain-inoculated and *mar1*Δ mutant strain-inoculated lungs displayed a diffuse but minimal pattern of acute inflammation (Fig. 1B). The only notable difference observed at this time point was that fungal cells were much more readily observed in WT strain-inoculated lungs than in *mar1*Δ mutant strain-inoculated lungs (Fig. 1B). This observation was supported by our previously published experiments in which *mar1*Δ mutant strain-inoculated lungs had a significant reduction in fungal burden compared to WT strain-inoculated lungs as early as 1 and 4 dpi (23). Additionally, unlike *mar1*Δ mutant cells, many WT cells had undergone Titan cell transition with cell body enlargement and highly expanded capsules, as previously described (30).

By 7 dpi, WT strain-inoculated mice began to show signs of fungal disease, but *mar1*Δ mutant strain-inoculated mice still appeared clinically healthy. At this time point, WT strain-inoculated lungs displayed numerous small foci of inflammation (mean diameter, 535 μm) that failed to contain all fungal cells (Fig. 1C and F; see Fig. S1 in the supplemental material). These foci of inflammation also occasionally displayed histologic hallmarks of early granuloma formation, including the presence of epithelioid macrophages (1, 2, 4) (Fig. 1C; Fig. S1). This nascent granulomatous inflammatory response has been reported previously in the C57BL/6 mouse background infected with the C. neoformans serotype D strain 52D (17). In contrast, *mar1*Δ mutant strain-inoculated lungs had few visible fungal cells and displayed a more uniform pattern of inflammation throughout the lungs at 7 dpi (Fig. 1C). These observations demonstrate that distinct characteristics of the *mar1*Δ mutant strain pathology emerge early in infection.

At 14 dpi, a time point in infection in which WT strain-inoculated mice began to succumb to fungal infection and *mar1*Δ mutant strain-inoculated mice still appeared healthy, WT cells, many of which were Titan cells, proliferated throughout the lungs, with a scattered, unorganized inflammatory response with mixed cell infiltrates (Fig. 1D; Fig. S1). Additionally, in these WT strain-inoculated mice, the number of observed granulomatous regions was reduced from 7 dpi because most nascent granulomatous regions had broken down, concurrent with accelerating clinical symptoms and imminent mortality (Fig. 1D and F). In contrast, *mar1*Δ mutant strain-inoculated lungs began to form granulomatous regions of inflammation by 14 dpi. Specifically, foci of inflammation (mean diameter, 866 μm) contained rare, small (non-Titan) fungal cells. The regions of the lung immediately surrounding these foci of inflammation were mostly normal appearing, without fungal or inflammatory cells (Fig. 1D and F). Additionally, these inflammatory foci contained hallmarks of granulomas, such as epithelioid macrophages surrounded by lymphocytes (1, 2, 4) (Fig. 1D). In contrast to WT strain-inoculated mice, *mar1*Δ mutant strain-inoculated mice displayed few infection-related symptoms at this time point.

For *mar1*Δ mutant strain-inoculated mice that survived to 40 dpi, we observed well-circumscribed foci of more mature granulomatous inflammation (mean diameter, 1,355 μm) containing fungal cells, multinucleated giant cells, and palisading epithelioid macrophages (Fig. 1E and F; Fig. S1). Additionally, fungal cells were not observed in lung tissue outside these granulomatous regions. Collectively, these observations suggest differences in the immune response in the context of WT and *mar1*Δ mutant strain infections. WT strain-inoculated mice showed a consistently robust mixed inflammatory response and Titan cell response with nascent granuloma formation during early stages of infection (7 dpi) (Fig. 1B and D to F; Fig. S1). As described previously (12), this response was ineffective and was quickly overcome by fungal growth, resulting in fungal proliferation throughout the lungs (14 dpi) (Fig. 1B to D). In contrast, *mar1*Δ mutant strain-inoculated mice showed a minimal inflammatory response, absent Titan cell formation, and minimal granulomatous inflammatory response during early stages of infection (7 dpi), with a more well-circumscribed granulomatous response in mice that survived to later time points in infection (40 dpi) (Fig. 1B to F; Fig. S1). These *mar1*Δ mutant strain-induced granulomatous regions appeared to be sufficient to contain most fungal proliferation, which was visualized using Movat staining to identify fungal cells with alcian blue (Fig. S2).

We found that *mar1*Δ mutant strain-inoculated mice that survived to 40 dpi remained healthy appearing up to 100 dpi. When these mice were sacrificed and cultured for viable C. neoformans cells, all isolated fungal colonies displayed phenotypic similarity to the initially inoculated *mar1*Δ mutant strain, including dry colony morphology on alkaline pH and nourseothricin (NAT) resistance (23) (Fig. S3). These observations indicate that the *mar1*Δ mutant strain can persist within murine lung granulomatous regions long term without causing any symptoms or signs of disease, creating a chronic state of fungal infection.

### Host GM-CSF signaling is required for the pulmonary granulomatous response.

**FIG 2:**
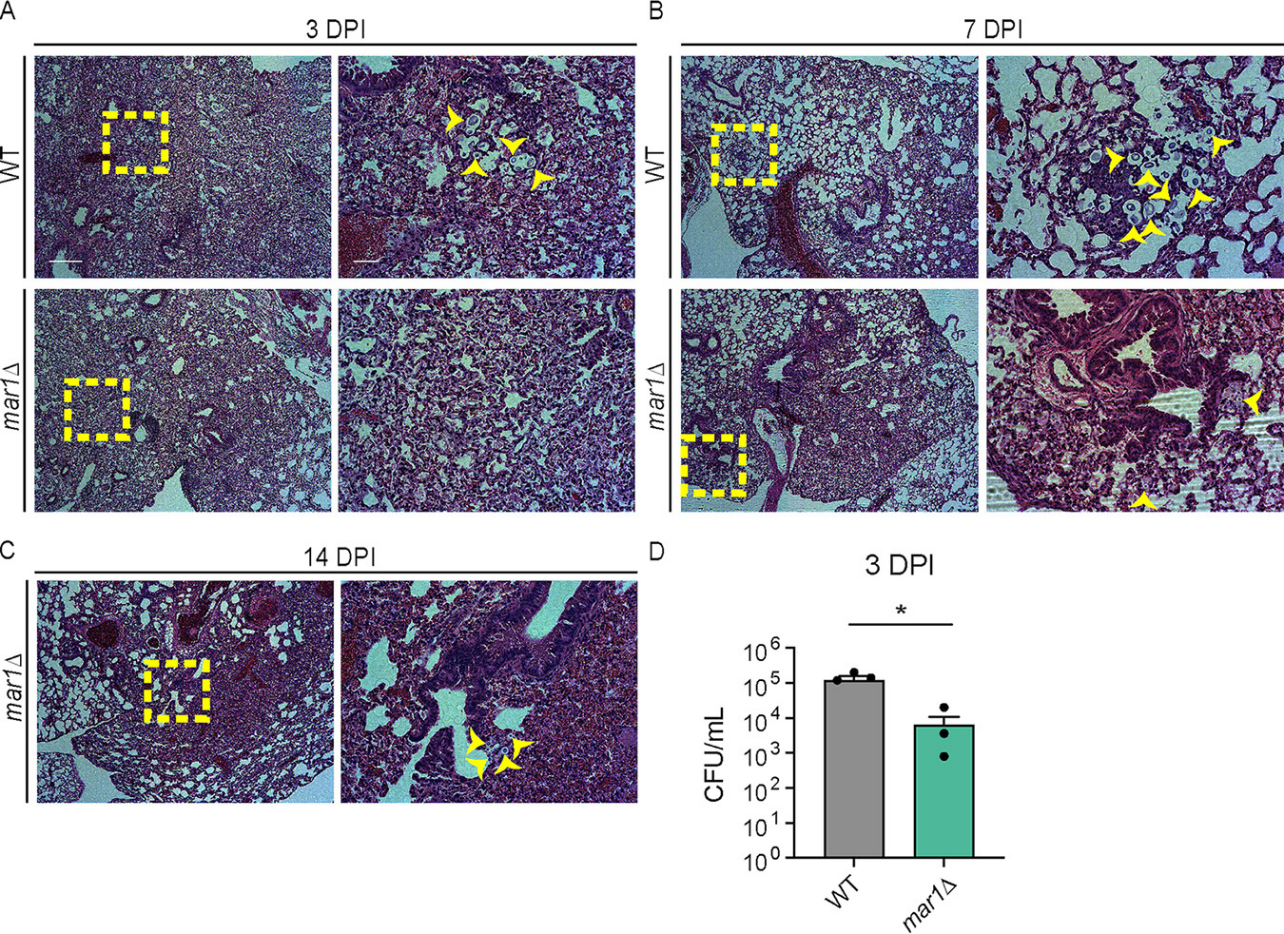
Contributions of GM-CSF signaling to the pulmonary granulomatous response. (A to C) The lungs of female (*n *= 2) (shown) and male (*n *= 2) (not shown) Csf2rb^−/−^ mice inoculated with 1 × 10^5^ cells of the WT strain or the *mar1*Δ mutant strain sacrificed at the predetermined time points of 3 (A), 7 (B), and 14 (C) dpi were harvested for histopathological analyses. Hematoxylin and eosin staining was utilized to visualize microscopic lung pathology (fungal cells [yellow arrowheads], inset [yellow boxes]). The 5× scale bar (left) is 250 μm, and the 10× scale bar (right) is 50 μm. (D) Pulmonary fungal burden of female (*n *= 2) and male (*n *= 2) Csf2rb^−/−^ mice inoculated with 1 × 10^5^ cells of the WT strain or the *mar1*Δ mutant strain sacrificed at 3 dpi was measured by quantitative cultures. Error bars represent the standard error of the mean (SEM). Statistical significance was determined using Student's *t* test (*, *P < *0.05).

We first explored host features that contribute to the granulomatous response in this model. Granulocyte-macrophage colony-stimulating factor (GM-CSF), a cytokine required for the maturation of myeloid cells, is required for granuloma formation in both mycobacterial (24–26) and cryptococcal (17) infections. We therefore hypothesized that GM-CSF signaling would also be necessary for the formation of pulmonary granulomatous regions in our model. To test this hypothesis, we assessed the progression of infections with the WT strain and the *mar1*Δ mutant strain in the Csf2rb^−/−^ mouse background, which is defective in GM-CSF signaling due to loss of the functional GM-CSF receptor. We inoculated Csf2rb^−/−^ mice using the inhalation route and harvested lungs for analysis throughout infection. Overall, a similar pattern of inflammation was observed between mice inoculated with the WT strain and mice inoculated with the *mar1*Δ mutant strain. We observed that well-defined pulmonary granulomatous regions were absent in Csf2rb^−/−^ mice infected with either strain at every tested time point (3, 7, and 14 dpi) (Fig. 2A to C; Fig. S4). Instead, the pattern of inflammation appeared poorly organized and diffuse throughout the entirety of the lungs infected with either fungal strain. Like the C57BL/6 infections, WT fungal cells were abundant throughout the lung, many of which appeared to be Titan cells, while *mar1*Δ mutant fungal cells were infrequently observed and did not appear to form Titan cells (Fig. 2A to C; Fig. S4). Pulmonary fungal burden assessed at 3 dpi confirmed that *mar1*Δ mutant strain-inoculated lungs had a significantly lower fungal burden, with a 10-fold reduction compared to WT strain-inoculated lungs, similar to what was observed previously in C57BL/6 infections (23) (Fig. 2D). These data demonstrate that GM-CSF signaling is required for the granulomatous response in both WT and *mar1*Δ mutant strain infections. However, because loss of GM-CSF signaling did not rescue the reduction of *mar1*Δ mutant strain fungal burden during early stages of infection, these data also suggest that GM-CSF signaling does not exclusively drive the impaired fitness of *mar1*Δ mutant cells in the murine lung.

### The *mar1*Δ mutant strain has a reduced fungal burden and induces an altered immune response *in vivo*.

We next explored fungal features that contribute to the granulomatous response in this model. We previously reported that in C57BL/6 mice, *mar1*Δ mutant strain-inoculated lungs displayed a significant decrease in fungal burden compared to WT strain-inoculated lungs, with a >10-fold decrease as early as 1 dpi (23). To determine whether chronic granulomatous inflammation was simply a product of a lower persisting fungal burden in *mar1*Δ mutant strain-inoculated lungs, we inoculated mice with 10-fold fewer cells (1 × 10^4^ CFU) than were used in previous experiments (1 × 10^5^ CFU). We observed similar histological patterns of lung inflammation with this lower inoculum as we did with the higher inoculum. Like our observations with the higher inoculum (Fig. 1; Fig. S1), murine lungs infected with the lower inoculum of the WT strain displayed robust fungal proliferation and a poorly organized inflammatory response by 14 dpi, demonstrating that a 10-fold reduction in the infecting dose was not sufficient to induce chronic granulomatous inflammation (Fig. S5A to D). Also similar to our prior experiments, by 21 dpi murine lungs infected with the lower inoculum of the *mar1*Δ mutant strain exhibited well-circumscribed regions of granulomatous inflammation with multinucleated giant cells and epithelioid macrophages, as well as rare fungal cells present only in these focal regions of inflammation (Fig. S5E to H). All WT strain-inoculated mice succumbed to fungal infection within 30 dpi with this lower inoculum, similar to the survival kinetics previously observed with the higher inoculum (23) (Fig. S5I). In contrast, *mar1*Δ mutant strain-inoculated mice displayed 90% survival at 60 dpi with this lower inoculum (Fig. S5I). Because this lower infecting dose resulted in similar histological features of granulomatous inflammation to the higher infecting dose, while maintaining a clearer distinction between uniformly lethal infections due to the WT strain and more indolent infections due to the *mar1*Δ mutant strain, we utilized this lower inoculum to examine the immune environment of the infected murine lung.

**FIG 3:**
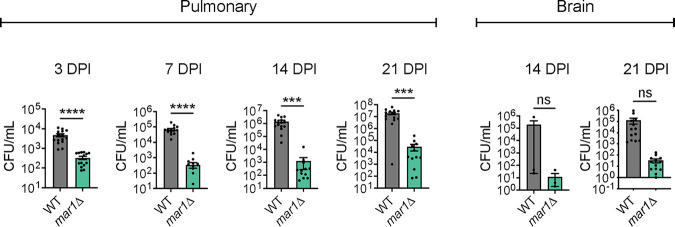
Fungal burden throughout infection. Pulmonary fungal burden of female C57BL/6 mice (*n *= 15) inoculated with 1 × 10^4^ cells of the WT strain or the *mar1*Δ mutant strain was measured by quantitative cultures throughout infection: 3, 7, 14, and 21 dpi. Brain fungal burden of female C57BL/6 mice (*n *= 15) inoculated with 1 × 10^4^ cells of the WT strain or the *mar1*Δ mutant strain was measured by quantitative cultures at 14 and 21 dpi. Error bars represent the SEM. Statistical significance was determined using Student's *t* test (***, *P < *0.001; ****, *P < *0.0001; ns, not significant).

We infected mice with this lower inoculum and assessed fungal burden and pulmonary cytokine and leukocyte responses at time points relevant to the progression of the observed granulomatous regions. At all tested time points (3, 7, 14, and 21 dpi), *mar1*Δ mutant strain-inoculated lungs had a significantly reduced fungal burden compared to WT strain-inoculated lungs. Specifically, *mar1*Δ mutant strain-inoculated lungs had a 10-fold reduction in fungal burden at 3 dpi, a 100-fold reduction in fungal burden at 7 dpi, and a >500-fold reduction in fungal burden at 14 and 21 dpi compared to WT strain-inoculated lungs (Fig. 3). These observations support the reduced number of *mar1*Δ mutant cells observed at these same time points in our histopathology analyses (Fig. 1B to D). As a result of the drastic reduction in pulmonary fungal burden throughout infection, we observed that the *mar1*Δ mutant strain also had attenuated dissemination to the brain (Fig. 3). Together, these observations indicate that the *mar1*Δ mutant strain has reduced fungal burden in the murine lung and brain over a range of infecting inocula.

**FIG 4:**
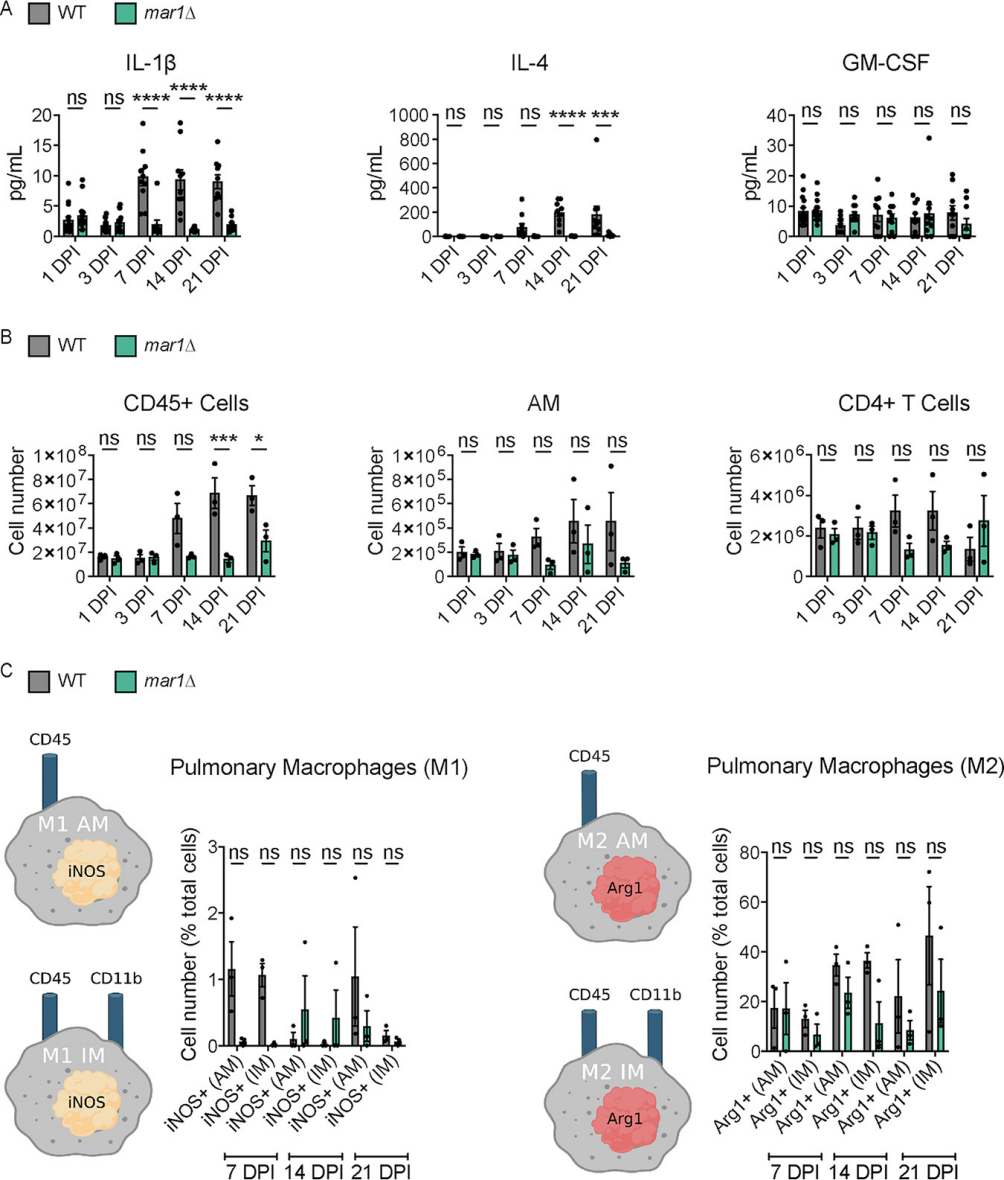
Pulmonary cytokine profile and leukocyte infiltrate associated with the granulomatous response. (A) Pulmonary cytokine responses of female C57BL/6 mice inoculated with 1 × 10^4^ cells of the WT strain or the *mar1*Δ mutant strain were measured using the Bio-Plex protein array system throughout infection: 1 (*n *= 15), 3 (*n *= 15), 7 (*n *= 10), 14 (*n *= 10), and 21 (*n *= 10) dpi. Error bars represent SEM. Statistical significance between strains at each time point was determined using two-way ANOVA (***, *P < *0.001; ****, *P < *0.0001; ns, not significant). Only a subset of data is shown; refer to Fig. S6 in the supplemental material for full analysis. (B) Pulmonary leukocyte infiltrates of female C57BL/6 mice inoculated with 1 × 10^4^ cells of the WT strain or the *mar1*Δ mutant strain were measured by flow cytometry throughout infection: 1, 3, 7, and 21 dpi. Data shown are the mean ± SEM of absolute cell numbers from three independent experiments (*n *= 3) performed using five mice per group per time point per experiment. Error bars represent SEM. Statistical significance between strains at each time point was determined using two-way ANOVA (*, *P < *0.05; ***, *P < *0.001; ns, not significant). Only a subset of data is shown; refer to Fig. S7 for full analysis. (C) Pulmonary macrophage activation of female C57BL/6 mice (*n *= 3) inoculated with 1 × 10^4^ cells of the WT strain or the *mar1*Δ mutant strain was measured by flow cytometry throughout infection: 7, 14, and 21 dpi. Inducible nitrogen oxide synthase (iNOS) was used as a marker for M1 macrophages, and arginase 1 (Arg1) was used as a marker for M2 macrophages. The percentages of total iNOS^+^ cells and Arg1^+^ cells are shown. Error bars represent the SEM. Log transformation was used to normally distribute the data for statistical analysis. Statistical significance between strains at each time point was determined using two-way ANOVA. AM, alveolar macrophage (CD45^+^ CD11b^−^); IM, interstitial macrophage (CD45^+^ CD11b^+^). (Created with BioRender.com).

Based on the differences in fungal burden observed between WT strain-inoculated and *mar1*Δ mutant strain-inoculated lungs, we hypothesized that the immune microenvironments within the lungs would also differ significantly. At early time points in infection, 1 and 3 dpi, we observed similar pulmonary cytokine and leukocyte profiles within WT strain-inoculated lungs and *mar1*Δ mutant strain-inoculated lungs (Fig. 4A and B; Fig. S6 and S7). Despite the significant reduction in *mar1*Δ mutant fungal burden at these early time points, the *mar1*Δ mutant strain induced cytokine and leukocyte responses comparable to those of the WT strain. As infection progressed to 7, 14, and 21 dpi, we observed marked reductions in multiple cytokines (including interleukin-1 β [IL-1β] and IL-4) and leukocytes (including CD45^+^ cells and alveolar macrophages) in *mar1*Δ mutant strain-inoculated lungs compared to WT strain-inoculated lungs (Fig. 4A and B; Fig. S6 and S7). While WT strain-inoculated lungs generally displayed increases in most tested cytokines and leukocytes as infection progressed, *mar1*Δ mutant strain-inoculated lungs generally displayed stable levels of most tested cytokines and leukocytes throughout the course of infection. This observation was further supported by our histopathological observations made at the same time points demonstrating localized regions of inflammation in *mar1*Δ mutant strain-inoculated lungs (Fig. 1C and D). These observations demonstrate that by mid to late time points in infection, the overall cytokine and leukocyte responses are reduced in *mar1*Δ mutant strain-inoculated lungs compared to WT strain-inoculated lungs, likely due to the progressive reduction in fungal burden present in *mar1*Δ mutant strain-inoculated lungs.

We further explored macrophage polarization at the same time points to determine whether the reduction in *mar1*Δ mutant strain fungal burden and the subsequent reduction in the pulmonary immune response were due to differences in macrophage activation (31). At each tested time point (7, 14, and 21 dpi), we observed that the *mar1*Δ mutant strain-inoculated lungs had a comparable number of or fewer classically activated (M1) and alternatively activated (M2) alveolar and interstitial macrophages compared to WT strain-inoculated lungs (Fig. 4C). These observations demonstrate that the *mar1*Δ mutant strain does not induce differential macrophage polarization causing the observed reductions in fungal burden and a more protective immune response. Collectively, these data suggest that the *mar1*Δ mutant strain-induced pulmonary granulomatous response appears to be a largely fungus-driven phenomenon. Despite reductions in fungal burden, the *mar1*Δ mutant strain induces a WT strain-like immune response early in infection. As infection matures and progresses, there is a relative decrease compared to WT strain-inoculated lungs in many cytokines and leukocytes infiltrating the *mar1*Δ mutant strain-inoculated lungs that corresponds with the progressive reduction in fungal burden and resulting fungal containment within granulomatous regions.

### The *mar1*Δ mutant strain is attenuated in the employment of various virulence factors.

**FIG 5:**
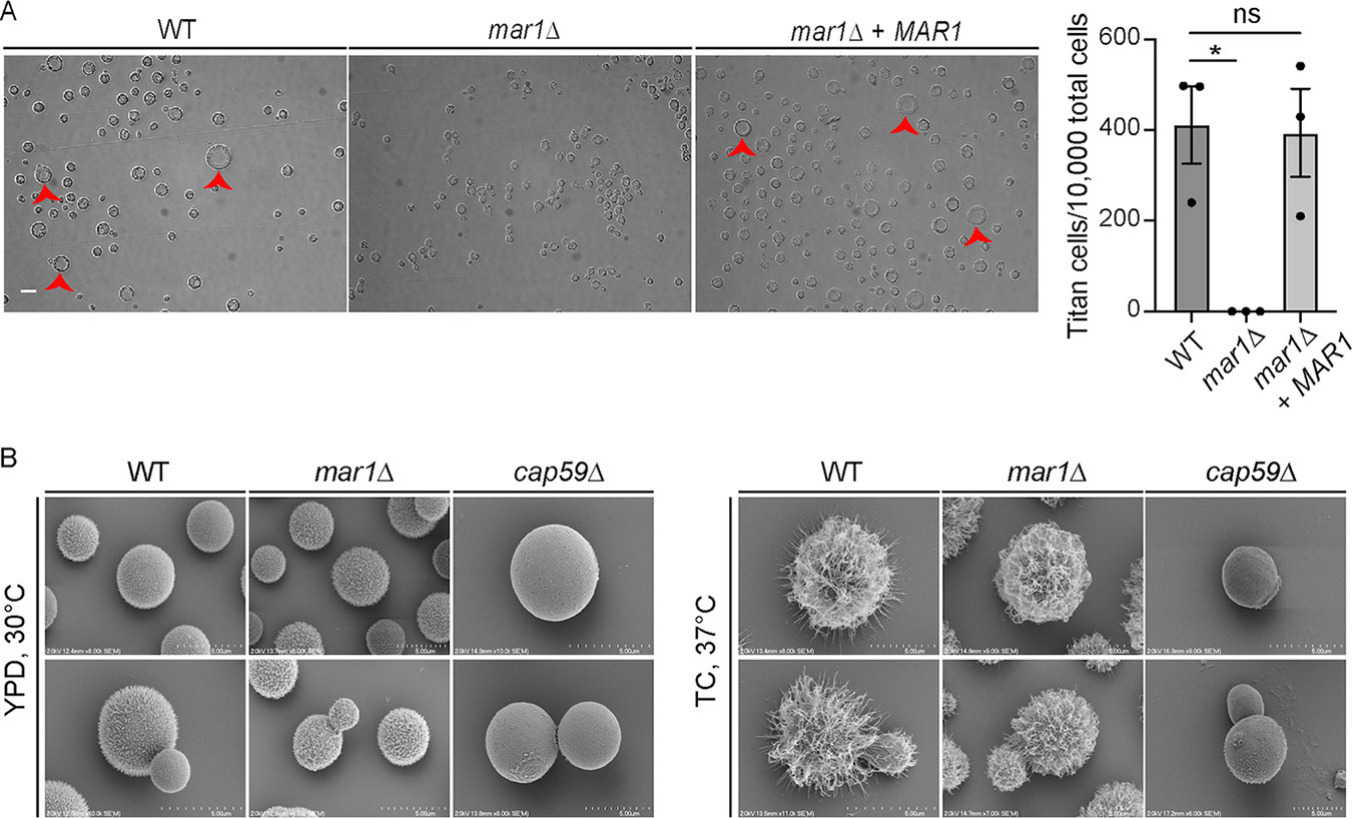
Pathogenesis-relevant virulence factor phenotypes of the *mar1*Δ mutant strain. (A) Titan cell formation was induced in the WT strain, the *mar1*Δ mutant strain, and the *mar1*Δ + *MAR1* complemented strain. Cells were pregrown in YNB medium at 30°C, and an OD_600_ of 0.001 was transferred to 10% HI-FBS in PBS incubated at 5% CO_2_ at 37°C for 96 h. Cells were imaged by DIC microscopy (Zeiss Axio Imager A1). Cell diameter was measured using Fiji, and cells with a diameter of >10 μm were considered Titan cells (red arrowheads). The number of Titan cells per 10,000 cells was calculated for each strain. A minimum of 400 cells were analyzed across three biological replicates (*n *= 3). Error bars represent the SEM. Statistical significance was determined using a one-way ANOVA (*, *P < *0.05; ns, not significant). The 63× scale bar is 10 μm. (B) The WT strain, the *mar1*Δ mutant strain, and the *cap59*Δ mutant strain were incubated in YPD medium at 30°C and CO_2_-independent medium (TC) at 37°C until saturation. Samples were subsequently fixed, mounted, dehydrated, and sputter coated. Samples were imaged with a Hitachi S-4700 scanning electron microscope to visualize capsule organization and elaboration.

We next explored specific phenotypes of the *mar1*Δ mutant strain that may drive the observed granulomatous response. In both human and murine infections, a subset of cryptococcal cells form enlarged Titan cells, an important virulence factor that suppresses fungal phagocytosis by host macrophages (30, 32). Using an established *in vitro* titanization assay (33), we observed that the *mar1*Δ mutant strain was unable to form Titan cells (Fig. 5A). This observation supported our histopathology experiments, in which Titan cells were absent in *mar1*Δ mutant strain-inoculated lungs. Additionally, we previously reported that the *mar1*Δ mutant strain is impaired in the attachment of the polysaccharide capsule, assessed by India ink staining, a defect that was rescued by complementation with the *MAR1* gene (23). We utilized high-resolution scanning electron microscopy (SEM) to more rigorously study the *mar1*Δ mutant strain capsule architecture. Under permissive growth conditions (yeast extract-peptone-dextrose [YPD] medium, 30°C), the capsule of the *mar1*Δ mutant strain was nearly indistinguishable from that of the WT strain, which contrasted starkly with the acapsular *cap59*Δ mutant strain (Fig. 5B). However, under capsule-inducing conditions (TC medium, 37°C), the *mar1*Δ mutant strain lacked the same degree of capsule fiber elongation observed in the WT strain, explaining the reduction in India ink exclusion previously reported for the *mar1*Δ mutant strain (23) (Fig. 5B). Collectively, these observations demonstrate that the *mar1*Δ mutant strain is defective in implementing some virulence factors, phenotypes which may contribute to the enhanced immunogenicity of this strain *in vivo*.

### The *mar1*Δ mutant strain displays cell cycle defects that result in a slow-growth phenotype and hypoxia resistance.

**FIG 6:**
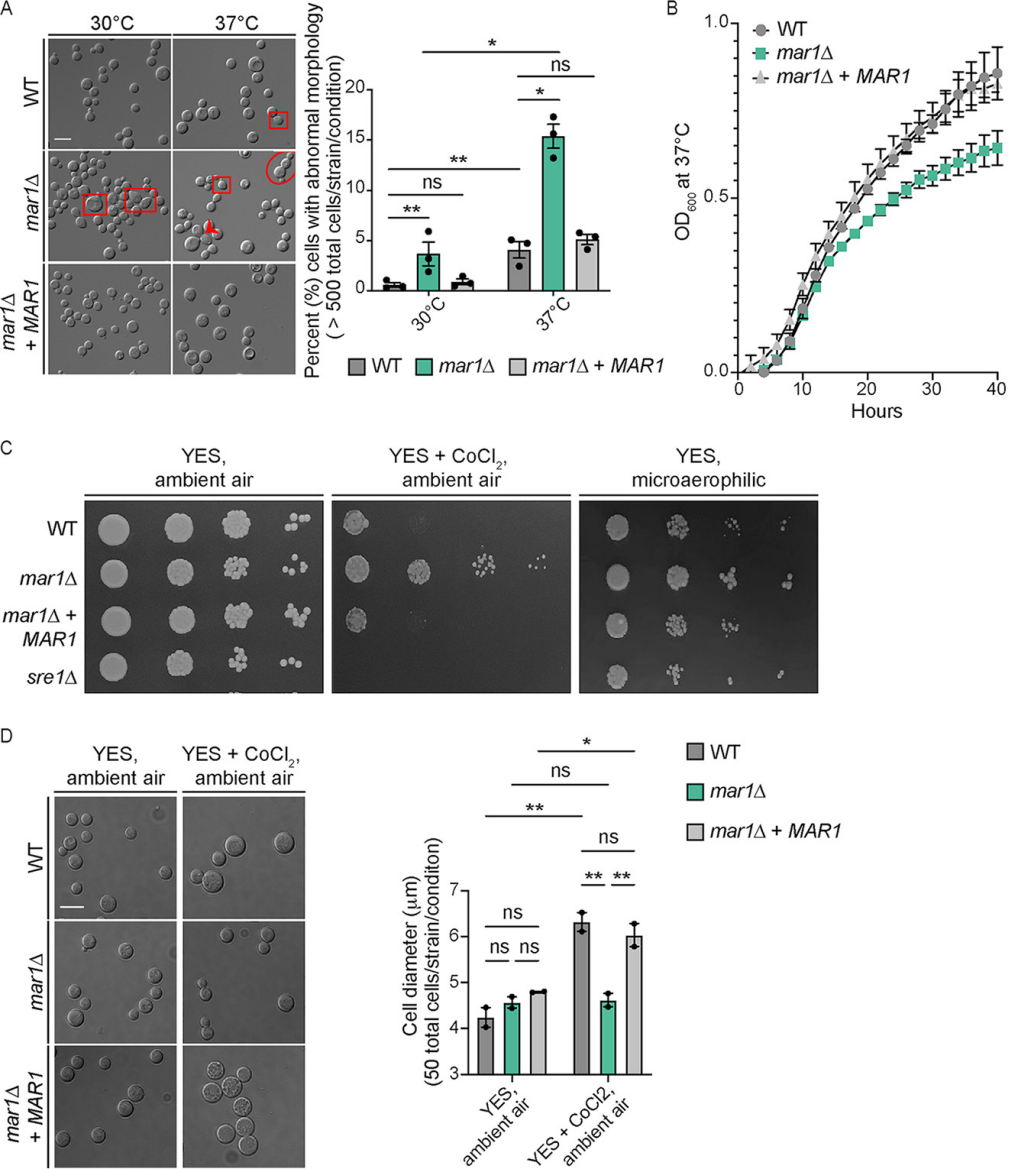
Slow-growth phenotypes of the *mar1*Δ mutant strain. (A) Morphological defects were analyzed in the WT strain, the *mar1*Δ mutant strain, and the *mar1*Δ + *MAR1* complemented strain through incubation in YPD medium at either 30°C or 37°C. Cells were imaged by DIC microscopy (Zeiss Axio Imager A1) and were subsequently visually inspected for morphological defects, such as elongated cells (red squares), wide bud necks (red arrowhead), and cytokinesis failure (red circle). The percentage of total cells displaying morphological defects was quantified for each strain at each temperature. A minimum of 500 cells were analyzed across three biological replicates (*n *= 3). Error bars represent the SEM. Log transformation was used to normally distribute the data for statistical analysis. Statistical significance between strains at each time point was determined using two-way ANOVA (*, *P < *0.05; **, *P < *0.01; ns, not significant). The 63× scale bar is 10 μm. (B) Growth of the WT strain, the *mar1*Δ mutant strain, and the *mar1*Δ + *MAR1* complemented strain was assessed in YPD medium at 37°C. Growth was tracked for 40 h and was measured by absorbance at OD_600_. The figure summarizes data across three biological replicates (*n *= 3). Error bars represent the SEM. (C) Hypoxia resistance was assessed by growth on YES medium supplemented with CoCl_2_ (0.7 mM) and in a microaerophilic chamber. Serial dilutions of the WT strain, *mar1*Δ mutant strain, *mar1*Δ + *MAR1* complemented strain, and *sre1*Δ mutant strain were spotted onto agar plates and incubated at 30°C. Results were compared to the same strains grown on YES medium under ambient air conditions. (D) The WT strain, *mar1*Δ mutant strain, and *mar1*Δ + *MAR1* complemented strain were incubated on YES medium with and without CoCl_2_ (0.7 mM) at 30°C under ambient air conditions. After 72 h of growth, cells were isolated and imaged by DIC microscopy (Zeiss Axio Imager A1). Cell diameter (in micrometers) was measured using Fiji. The average cell diameter was quantified for each strain under each condition. A minimum of 50 cells were analyzed across two biological replicates (*n *= 2). Error bars represent the SEM. Statistical significance was determined using a two-way ANOVA (*, *P < *0.05; **, *P < *0.01; ns, not significant). The 63× scale bar is 10 μm.

Both Titan cell formation (33, 34) and polysaccharide capsule elaboration (35–37) are mediated by the cell cycle. To explore cell cycle progression in the *mar1*Δ mutant strain background, we observed *mar1*Δ mutant cell morphology during the logarithmic growth phase. When incubated at the permissive temperature of 30°C, the *mar1*Δ mutant strain displayed an increased incidence of cytokinesis defects (such as elongated cells, cells with wide bud necks, and cells that fail to complete cytokinesis), compared to both the WT strain and the *mar1*Δ + *MAR1* complemented strain (Fig. 6A). The frequency of these cytokinesis defects was significantly enhanced at the host physiological temperature of 37°C (Fig. 6A). We next determined the impact of these defects on the growth kinetics of the *mar1*Δ mutant strain. We observed that the *mar1*Δ mutant strain displayed a reduction in growth during logarithmic phase at 37°C, compared to both the WT strain and the *mar1*Δ + *MAR1* complemented strain (Fig. 6B). These data demonstrate that the *mar1*Δ mutant strain has a slow-growth phenotype at mammalian body temperature, a phenotype that is likely driven in part by impaired cell cycle progression resulting in cytokinesis defects.

Cell cycle regulation is also known to be related to fungal adaptation to hypoxia (38–40). Because C. neoformans is an obligate aerobe, WT fungal cells undergo G_2_ arrest in response to hypoxia (41, 42). We assessed the ability of the *mar1*Δ mutant strain to grow in an environment with reduced oxygen availability by observing growth in the presence of CoCl_2_ and in a microaerophilic chamber. In both cases, we observed that the *mar1*Δ mutant strain displayed enhanced growth compared to the WT strain and the *mar1*Δ + *MAR1* complemented strain (Fig. 6C). In these assays, the CoCl_2_- and hypoxia-sensitive *sre1*Δ mutant strain was used as a control (43) (Fig. 6C). We then isolated the cells grown in the presence of CoCl_2_ to examine cell morphology. Consistent with previous literature that has reported that C. neoformans arrests in an unbudded G_2_ state in response to hypoxia (41, 42), we observed that the WT strain and the *mar1*Δ + *MAR1* complemented strain displayed a significant increase in cell size when incubated in the presence of CoCl_2_ (Fig. 6D). However, the *mar1*Δ mutant strain did not display this increase in cell size in response to CoCl_2_ (Fig. 6D). Collectively, these observations demonstrate that the *mar1*Δ mutant strain has altered cell cycle dynamics and relative resistance to hypoxia, features that potentially contribute to changes in microbial persistence during infection.

## DISCUSSION

Here, we report and characterize the host response to a chronic C. neoformans lung infection, one distinguished by sustained regions of granulomatous inflammation. Using the inhalation route of infection in C57BL/6 mice, we observed a granulomatous response in infections due to both the WT and *mar1*Δ mutant strains. However, the appearance, development, and maintenance of these granulomatous regions differed significantly. In WT infections, small, immature granulomatous regions formed early in infection. As infection progressed, these nascent granulomatous regions began to degenerate, leading to fungal proliferation throughout the lungs, fungal dissemination to the brain, and eventually murine death. This type of early, immature granulomatous response has been observed previously in murine infections with other C. neoformans WT strains (16, 17). In contrast, in *mar1*Δ mutant strain infections we observed chronic pulmonary granulomatous regions that developed over several weeks in the absence of overt clinical symptoms. These granulomatous regions differed from those induced by the WT strain because they appeared later in infection, were typically larger, and were more circumscribed. Furthermore, we observed that *mar1*Δ mutant strain-induced granulomatous regions were maintained throughout infection, from 14 dpi to as late as 100 dpi. Collectively, the *mar1*Δ mutant strain displays a unique interaction with the host characterized by granulomatous inflammation and chronic infection.

We confirmed the role of GM-CSF as a host driver of cryptococcal granuloma formation. In our model, GM-CSF was modestly elevated in *mar1*Δ mutant strain-inoculated lungs at 1, 3, and 14 dpi. This increased GM-CSF production may be a result of increased Dectin-1 activation by the *mar1*Δ mutant strain. We previously reported that the *mar1*Δ mutant strain is partially recognized by the pathogen recognition receptor Dectin-1, likely due its increased exposed surface β-glucan and chitin (23). Dectin-1 has been shown to be required for normal GM-CSF production in murine macrophages (44). Additionally, GM-CSF production is known to result in an increase in Dectin-1 expression by murine macrophages (44, 45). Here, we found that the granulomatous response in this model is dependent on GM-CSF signaling, as granulomatous regions were absent in Csf2rb^−/−^ mouse background infections with either the WT or *mar1*Δ mutant strains. These results were expected because GM-CSF plays a significant role in controlling infections due to both Cryptococcus gattii and C. neoformans, as individuals with GM-CSF autoantibodies are unusually susceptible to cryptococcal infection (46–48). Furthermore, previous work in both mycobacterial (24–26) and cryptococcal (17) infections has demonstrated that GM-CSF signaling is required for granuloma formation, likely due to its requirement for macrophage recruitment to the lung during early stages of infection. Our model enables further exploration of the requirement of GM-CSF for granuloma maintenance. For example, future experiments could introduce GM-CSF antibodies into *mar1*Δ mutant strain-inoculated mice to determine whether GM-CSF is required for the maintenance of *mar1*Δ mutant strain-induced granulomatous regions. Furthermore, WT strain infections could be supplemented with exogenous GM-CSF to determine whether increased GM-CSF can help maintain WT strain-induced granulomatous regions.

We characterized multiple fungal features associated with chronic inflammation in this model. We observed that throughout the course of infection, *mar1*Δ mutant strain-inoculated lungs had a significantly lower fungal burden than WT strain-inoculated lungs. Despite this decrease in fungal burden, *mar1*Δ mutant strain-inoculated lungs induced WT strain-like pulmonary cytokine and leukocyte responses during early stages of infection. As infection progressed, WT strain-inoculated lungs continued to display marked increases in pulmonary cytokines and leukocytes, while *mar1*Δ mutant strain-inoculated mice displayed more stable levels of pulmonary cytokines and leukocytes. Previous work has implicated classically activated macrophage polarization in enhanced antifungal activity of macrophages (49–51). We found that *mar1*Δ mutant strain-inoculated lungs had a comparable number of or fewer (depending on the time point) classically activated (M1) and alternatively activated (M2) macrophages compared to WT strain-inoculated lungs, suggesting that differential polarization of macrophages did not contribute to the reduced fungal burden and associated immune response in *mar1*Δ mutant strain-inoculated lungs.

We also characterized multiple *mar1*Δ mutant strain phenotypes that may contribute to the granulomatous response and fungal persistence in this model. Titan cell formation is a well-characterized, Cryptococcus-specific persistence mechanism that suppresses fungal phagocytosis by host macrophages (30, 32). Results from an established *in vitro* titanization assay (33), as well as our histopathological observations, demonstrated that the *mar1*Δ mutant strain was impaired in the production of Titan cells. As a result, Titan cell transitions do not explain the persistence of the *mar1*Δ mutant strain in the murine lung. We also observed that the *mar1*Δ mutant strain was altered in the implementation of another important virulence factor, the polysaccharide capsule. Although the *mar1*Δ mutant strain had a similar basal level of capsule to the WT strain, it was unable to extend its capsule fibers to the level of the WT strain in response to capsule-inducing signals. In addition to the previously reported defects in the *mar1*Δ mutant strain cell wall (23), these virulence factor defects likely contribute to the enhanced immunogenicity of the *mar1*Δ mutant strain.

The expression of many virulence factors is known to be mediated by the cell cycle (37). Furthermore, recent work has proposed that C. neoformans undergoes a unique cell cycle *in vivo*, the “stress cell cycle,” that regulates the employment of various virulence factors (52). Titan cells are enlarged and polyploid cells that form in both human and mouse lungs during infection (30, 32). This polyploidization and concomitant cell body enlargement is negatively regulated by the transcription factor Usv101, which acts downstream of the cell cycle regulator Swi6 (33, 34). Furthermore, recent work has found that the Cln1 cyclin contributes to Titan cell formation by regulating DNA replication and cell division after G_2_ arrest *in vivo* (52). Similarly, capsule elongation is also regulated by the cell cycle, with the majority of capsule elongation occurring in the G_1_ phase of the cell cycle (36). The dysregulation of these cell-cycle-mediated virulence factors suggested that the *mar1*Δ mutant strain harbored cell cycle defects.

We indeed observed that the *mar1*Δ mutant strain displayed a marked increase in cytokinesis defects compared to the WT strain, particularly at 37°C, leading to a decreased growth rate. We furthermore observed that the *mar1*Δ mutant strain had enhanced growth in hypoxia, potentially because it did not arrest in an unbudded G_2_ state like the WT strain. In various cell types, including stem cells (53), tumor cells (54), bacteria (55), and fungi (39), a reduction in growth rate is required for survival in the presence of hypoxia. It is possible that its inherent decreased growth rate predisposed the *mar1*Δ mutant strain to growth in a hypoxic environment. The mammalian environment is known to limit oxygen availability to invading microorganisms, as a stressor used to contain microbial proliferation (38). This important resource is likely even further restricted within the pulmonary granuloma, which is known to have suboptimal oxygen levels in the context of mycobacterial infection (56). Recent work by the Alanio laboratory has demonstrated that cryptococcal dormancy can be induced by a combination of nutrient and oxygen deprivation (40, 57). Furthermore, the Dromer laboratory has found that dormant cryptococcal cells are characterized by reduced metabolic activity and delayed growth (39). With these observations in mind, it is possible that the slow-growth and hypoxia resistance phenotypes of the *mar1*Δ mutant strain favor its containment and persistence within granulomatous regions in the model described here. Further work will be required to determine whether these phenotypes are necessary and/or sufficient for fungal containment and persistence within granulomatous regions.

Collectively, these observations suggest that the *mar1*Δ mutant strain-induced granulomatous response is largely a fungus-driven phenomenon. Murine infections with the *mar1*Δ mutant strain are characterized by reduced fungal burden, likely due to the low growth rate of the *mar1*Δ mutant strain, and an altered host immune response, likely due to the virulence factor attenuation of the *mar1*Δ mutant strain. At early time points in infection, the immunogenic *mar1*Δ mutant strain induces a WT strain-like pulmonary immune response despite a marked reduction in fungal burden. As infection progresses, the WT strain proliferates rapidly throughout the lungs, resulting in a robust pulmonary immune response in WT strain-inoculated lungs that is associated with murine mortality, while the slow-growing *mar1*Δ mutant strain remains contained within granulomatous regions. Using these approaches, we have defined a detailed timeline of the murine granulomatous response, in both WT strain and *mar1*Δ mutant strain infections, and characterized multiple fungal factors that contribute to this response and long-term fungal persistence (see Fig. S8 in the supplemental material).

The Del Poeta laboratory has developed a well-characterized murine pulmonary granulomatous response model of cryptococcal disease using the *gcs1*Δ mutant strain. From the fungal perspective, the *gcs1*Δ mutant strain lacks the membrane sphingolipid glucosylceramide, making it an obligate intracellular pathogen and, as a result, completely avirulent in a murine inhalation model, the route of infection that most closely replicates the course of human infection (18, 19). It is noteworthy that the *gcs1*Δ and *mar1*Δ mutant strains were constructed in the same WT strain background, and as a result, these two mutant strains are comparable and could potentially be used together to explore the complex characteristics of granuloma formation. For example, both strains display cell cycle defects in the presence of physiological stress: the *gcs1*Δ mutant strain arrests at alkaline pH (18), and the *mar1*Δ mutant strain displays cytokinesis defects at 37°C. These similarities suggest that a slow-growth phenotype in the host environment may favor fungal containment within regions of granulomatous inflammation. Both strains likely also exist frequently within macrophages *in vivo*, as the *gcs1*Δ mutant strain is an obligate intracellular pathogen (18) and the *mar1*Δ mutant strain has phenotypes, like reduced Titan cell formation and polysaccharide capsule extension, that predispose fungal cells to phagocytosis by macrophages. Virulence potential is a notable difference between the strains. The *gcs1*Δ mutant strain is unable to initiate infection and disease via the inhalation route of infection (18), categorizing *GCS1* as a disease initiation factor (58). In contrast, the *mar1*Δ mutant strain can establish infection and cause fatal disease in nearly half of the mice infected with 1 × 10^5^ CFU (23), making *MAR1* a disease progression factor (58). This may be related to the fact that *GCS1* orthologs are found in many pathogenic fungi (18), while *MAR1* appears to be a Cryptococcus-specific gene (23). These contrasting features suggest that the granulomatous response is a highly complex process that relies on the interplay between many host and fungal factors.

From the host perspective, *gcs1*Δ mutant strain-induced granuloma formation requires host sphingosine kinase 1–sphingosine 1-phosphate (SK1-S1P) signaling (20, 21). Most recently, the Del Poeta laboratory has applied this model to explore cryptococcal reactivation. Mimicking human disease, *gcs1*Δ mutant cells become reactivated from granulomas and disseminate upon immunosuppression with the multiple sclerosis therapeutic FTY720, which suppresses SK1-S1P signaling (22). This model has enabled the first murine reactivation studies of cryptococcal infection. Future work with the *mar1*Δ mutant strain-induced granulomatous response could similarly explore reactivation in the context of immunosuppression, to better understand the typical course of cryptococcal disease in humans. One of the populations most vulnerable to cryptococcal reactivation includes untreated HIV/AIDS patients (7). In our leukocyte infiltrate analyses, we observed that *mar1*Δ mutant strain-inoculated lungs had an enhanced CD4^+^ T cell response compared to WT strain-inoculated lungs at 21 dpi. This observation is particularly striking because *mar1*Δ mutant strain-inoculated lungs had a decreased or equivalent response compared to WT strain-inoculated lungs for all other leukocytes tested at this time point. CD4^+^ T cells are present in pulmonary granulomas of immunocompetent humans (59). Furthermore, CD4^+^ T cells border the periphery of pulmonary granulomas in HIV^+^ individuals receiving antiretroviral therapy, but they are lost in individuals with advanced HIV/AIDS, suggesting that CD4^+^ lymphocytes may be involved in granuloma maintenance (2, 59). By inducing CD4^+^ T cell depletion, and as a result mimicking the HIV/AIDS disease state, we could probe the role of CD4^+^ T cells in the maintenance of granulomatous regions in this model. Following immunosuppression, we can observe *mar1*Δ mutant strain-inoculated mice to track the breakdown of granulomatous regions and fungal proliferation with the same approaches used here. Considering both the fungal and host drivers of the granulomatous response outlined here, this model harbors features that make it unique from other existing cryptococcal infection models. Together with the conventional primary murine infection model, chronic murine infection models will advance our understanding of cryptococcal disease progression and define fungal features important for persistence in the human host.

## MATERIALS AND METHODS

### Strains, media, and growth conditions.

All strains used in this study were generated in the C. neoformans var. *grubii* H99 (*MAT*α) (13) background and are included in Table 1. Strains were maintained on yeast extract-peptone-dextrose (YPD) medium (1% yeast extract, 2% peptone, 2% dextrose, and 2% agar for solid medium). Unless otherwise indicated, strains were incubated at 30°C.

**TABLE 1:** Fungal strains used in this study

Strain	Genotype	Reference
H99	*MAT*α	13
MAK1	*MAT*α *mar1*Δ::*NAT*	23
MAK11	*MAT*α *mar1*Δ::*NAT + MAR1-NEO*	23
cap59	*MAT*α *cap59Δ*::*NEO*	63
HEB6	*MAT*α *sre1Δ*::*NEO*	43

### Histology analyses.

The murine inhalation model of cryptococcosis was exclusively used in this study (60). For initial histological examination, C57BL/6 female mice were acquired from Charles River Laboratories. Mice were anesthetized with 2% isoflurane utilizing a rodent anesthesia device (Eagle Eye Anesthesia, Jacksonville, FL) and were infected via the intranasal route with 1 × 10^5^ CFU of either the wild-type (WT) (H99) or the *mar1*Δ mutant (MAK1) strain. Mice were sacrificed at predetermined time points (3, 7, 14, and 40 days postinoculation [dpi]) by CO_2_ inhalation followed by an approved secondary method of euthanasia. Lungs were perfused with and stored in 10% neutral buffered formalin. Lungs were subsequently paraffin embedded, sectioned, mounted, and stained with hematoxylin and eosin and/or Movat by the Duke University School of Medicine Research Immunohistochemistry Shared Resource. Hematoxylin- and eosin-stained slides were used to identify granulomatous regions and characteristic features of granulomatous inflammation (e.g., multinucleated giant cells and epithelioid macrophages). Granulomatous regions of inflammation were quantified from one slide from three mice per strain per time point. Large cells with multiple nuclei were designated multinucleated giant cells. Mononuclear cells with an ovoid nucleus, dispersed chromatin, and eosinophilic cytoplasm were designated epithelioid macrophages. Movat-stained slides were used to more clearly visualize fungal cells within the lung tissue. All slides were reviewed in a blind manner by a pathologist (J.M.C.) with expertise in pulmonary pathology, including nonneoplastic and inflammatory lung disease.

To determine the role of GM-CSF signaling in the granulomatous response in this model, male and female Csf2rb^−/−^ mice (The Jackson Laboratory, no. 005940) were infected via the intranasal route as described above with 1 × 10^5^ CFU of either the WT (H99) or the *mar1*Δ mutant (MAK1) strain. Lungs were prepared as described above, with a few alterations. Mice were sacrificed at the predetermined time points of 3, 7, and 14 dpi by CO_2_ inhalation followed by an approved secondary method of euthanasia, and lungs were perfused with phosphate-buffered saline (PBS). The right lung was stored in 10% neutral buffered formalin for future histopathology preparation as described above, while the left lung was used for fungal burden quantification analyses as described below.

### Mouse isolate recovery and phenotypic characterization.

C57BL/6 female mice acquired from Charles River Laboratories were infected via the intranasal route as described above with 1 × 10^5^ CFU of either the WT (H99) or the *mar1*Δ mutant (MAK1) strain. At 61 dpi and 100 dpi, mice were sacrificed by CO_2_ inhalation followed by an approved secondary method of euthanasia. The lungs were removed and homogenized in 1 mL of sterile PBS as previously described (61). Single fungal colonies were subcultured onto YPD agar medium supplemented with chloramphenicol and subsequently frozen in separate wells of 96-well plates at −80°C. Isolated fungi were stamped onto YPD agar medium incubated at 30°C, YPD agar medium incubated at 37°C, YPD agar medium supplemented with nourseothricin (NAT) (100 μg/mL) incubated at 30°C, and YPD agar medium buffered (150 mM HEPES) to pH 8.15 and incubated at 30°C. All plates were imaged daily. Mouse isolates were determined to be *mar1*Δ mutant strain isolates based on growth on YPD+NAT medium and dry colony morphology on YPD medium at pH 8.15 (23). The original WT (H99) and *mar1*Δ mutant (MAK1) strains were included on each plate as controls.

### Murine survival studies.

C57BL/6 female mice acquired from Charles River Laboratories were infected via the intranasal route as described above with 1 × 10^4^ CFU of either the WT (H99) or the *mar1*Δ mutant (MAK1) strain. Mice were monitored over the course of 60 days and sacrificed based on clinical endpoints that predict mortality. Statistically significant differences between survival curves were determined by log rank test with Bonferroni correction (GraphPad Prism).

### Fungal burden quantification.

Infected mice were euthanized at predetermined time points by CO_2_ inhalation followed by an approved secondary method of euthanasia, and lung tissues and/or brain tissues were excised. Tissues were then homogenized in 1 mL of sterile PBS as previously described (61), followed by culture of 10-fold dilutions of each homogenate on YPD agar medium supplemented with chloramphenicol. CFU were enumerated following incubation at 30°C for 48 h. Statistical significance was determined using Student's *t* test (GraphPad Software, San Diego, CA).

### Pulmonary cytokine analyses.

C57BL/6 female mice acquired from Charles River Laboratories were infected via the intranasal route as described above with 1 × 10^4^ CFU of either the WT (H99) or the *mar1*Δ mutant (MAK1) strain. Mice were euthanized at predetermined time points by CO_2_ inhalation followed by cervical dislocation. Cytokine levels within the lung homogenates of infected mice were analyzed using the Bio-Plex protein array system (Luminex-based technology; Bio-Rad Laboratories, Hercules, CA). Briefly, lung tissues were excised and homogenized in 1 mL ice-cold sterile PBS. An aliquot (50 μL) was taken to quantify the pulmonary fungal burden, and an antiprotease buffer solution (1 mL) containing PBS, protease inhibitors, and 0.05% Triton X-100 was added to the homogenate. Samples were then clarified by centrifugation (3,500 rpm) for 10 min. Supernatants from pulmonary homogenates were assayed for the presence of IL-1α, IL-1β, IL-2, IL-3, IL-4, IL-5, IL-6, IL-9, IL-10, IL-12 (p40), IL-12 (p70), IL-13, IL-17, KC (CXCL1), monocyte chemoattractant protein (MCP-1) (CCL2), macrophage inflammatory protein 1α (MIP-1α) (CCL3), MIP-1β (CCL4), RANTES (CCL5), eotaxin (CCL11), gamma interferon (IFN-γ), tumor necrosis factor alpha (TNF-α), granulocyte-macrophage colony-stimulating factor (GM-CSF), and granulocyte colony-stimulating factor (G-CSF) according to the manufacturer’s instructions. Statistical significance between strains at each time point was determined using two-way analysis of variance (ANOVA) and the Tukey-Kramer test (GraphPad Software, San Diego, CA).

### Pulmonary leukocyte isolation.

C57BL/6 female mice acquired from Charles River Laboratories were infected via the intranasal route as described above with 1 × 10^4^ CFU of either the WT (H99) or the *mar1*Δ mutant (MAK1) strain. Mice were euthanized at predetermined time points by CO_2_ inhalation followed by cervical dislocation. Lungs of infected mice were excised and processed as previously described (61). Briefly, lungs were digested enzymatically at 37°C for 30 min in 10 mL digestion buffer (RPMI 1640 and 1 mg/mL collagenase type IV [Sigma-Aldrich, St. Louis, MO]) with intermittent (every 10 min) stomacher homogenizations. The digested tissues were then successively filtered through sterile 70- and 40-μm-pore nylon filters (BD Biosciences, San Diego, CA) to enrich for leukocytes, and the cells were then washed three times with sterile Hanks’ balanced salt solution (HBSS). Erythrocytes were lysed by incubation in NH_4_Cl buffer (0.859% NH_4_Cl, 0.1% KHCO_3_, 0.0372% Na_2_EDTA [pH 7.4]; Sigma-Aldrich) for 3 min on ice followed by a 2-fold excess of sterile PBS.

### Flow cytometry analyses.

Pulmonary leukocytes were isolated from mice infected as described above with 1 × 10^4^ CFU of either the WT (H99) or the *mar1*Δ mutant (MAK1) strain. Standard methodology was employed for the direct immunofluorescence of pulmonary leukocytes (61, 62). Briefly, in 96-well U-bottom plates, 100 μL containing 1 × 10^6^ cells in PBS were incubated with yellow Zombie viability dye (1:1,000 dilution; catalog no. 423104, Biolegend, San Diego, CA) for 15 min at room temperature followed by washing in fluorescence-activated cell sorter (FACS) buffer. Cells were then incubated with Fc block (1:500 dilution [catalog no. 553142, clone 2.4G2; BD Biosciences]) diluted in FACS buffer for 5 min to block nonspecific binding of antibodies to cellular Fc receptors. Cells were then incubated with fluorochrome-conjugated antibodies in various combinations to allow for multistaining for 30 min at 4°C. Cells were washed three times with FACS buffer and fixed in 200 μL of 2% ultrapure formaldehyde (Polysciences, Warrington, PA) diluted in FACS buffer (fixation buffer). Fluorescence minus one (FMO) controls or cells incubated with either FACS buffer alone or single fluorochrome-conjugated antibodies were used to determine positive staining and spillover/compensation calculations, and background fluorescence was determined with FlowJo v.10.8 software (FlowJo, LLC, Ashland, OR). Raw data were collected with a Cell Analyzer LSRII (BD Biosciences) using BD FACSDiva v8.0 software at the University of North Texas Health Sciences Center (UNTHSC) Flow Core, and compensation and data analyses were performed using FlowJo v.10.8 software. Cells were first gated for lymphocytes (SSC-A versus FSC-A) and singlets (FSC-H versus FSC-A). The singlets gate was further analyzed for the uptake of live/dead yellow stain to determine live versus dead cells. From live cells, cells were gated on CD45^+^ cell expression. For data analyses, 100,000 events (cells) were evaluated from a predominantly leukocyte population identified by back gating from CD45^+^ stained cells. Statistical significance between strains at each time point was determined using two-way analysis of variance (ANOVA) and the Tukey-Kramer test (GraphPad Software, San Diego, CA).

### Macrophage activation analyses.

Intracellular staining of markers of macrophage activation was performed as described previously (62). Leukocytes isolated from mice infected as described above with 1 × 10^4^ CFU of either the WT (H99) or the *mar1*Δ mutant (MAK1) strain were incubated with cell stimulation cocktail (eBioscience, catalog no. 00-4970-03) according to the manufacturer’s recommendation and incubated at 37°C in 5% CO_2_ in cRPMI for 2 h in a six-well plate. GolgiPlug (1:100 dilution, brefeldin A; catalog no. 51-2301KZ, BD Biosciences) was added according to the manufacturer’s recommendations, and the mixture was incubated for an additional 4 h (6 h total). Cells were washed with PBS and stained with yellow Zombie viability dye in PBS at room temperature in the dark for 15 min. Cells were then washed with FACS buffer and incubated with Fc block (BD Biosciences) diluted in FACS buffer for 5 min. For nitric oxide (inducible nitric oxide synthase [iNOS]) and arginase 1 (Arg1) production in macrophages, cells were stained for surface markers CD45, CD11b, CD64, F4/80, and CD24, and incubated at 4°C for 30 min. Cells were then washed and fixed with 2% ultrapure formaldehyde (Polysciences, Warrington, PA) for 20 min. Subsequently, cells were washed with 0.1% saponin buffer and stained with antibodies for iNOS and Arg1 for 30 min at 4°C. Finally, cells were washed with saponin buffer and fixed with 2% ultrapure formaldehyde. Samples were processed using a Cell Analyzer LSRII (BD Biosciences) using BD FACSDiva v.8.0 software at the UNTHSC Flow Core, and 100,000 events were collected for analysis using FlowJo v.10.8 software. Statistical significance between strains at each time point was determined using two-way analysis of variance (ANOVA) and the Tukey-Kramer test (GraphPad Software, San Diego, CA).

### Titan cell assay and quantification.

A previously described *in vitro* titanization assay was used here (33). In brief, the WT (H99), *mar1*Δ mutant (MAK1), and *mar1*Δ + *MAR1* (MAK11) strains were incubated for 18 h at 30°C at 150 rpm in 5 mL yeast nitrogen base (YNB) without amino acids plus 2% glucose prepared according to the manufacturer’s instructions. Cultures were washed six times with PBS. An optical density at 600 nm (OD_600_) of 0.001 for each strain was transferred to 5 mL 10% heat-inactivated fetal bovine serum (HI-FBS) in PBS and incubated at 37°C with 5% CO_2_ for 96 h. Cells were imaged by differential interference contrast (DIC) microscopy using a Zeiss Axio Imager A1 microscope equipped with an Axio‐Cam MRm digital camera. Cell diameter was measured using the ImageJ software (Fiji), and cells with a diameter of >10 μm were considered Titan cells. A minimum of 400 cells were analyzed across three biological replicates for each fungal strain. Statistical significance was determined using one-way analysis of variance (ANOVA) and the Tukey-Kramer test (GraphPad Software, San Diego, CA).

### SEM polysaccharide capsule visualization.

The WT (H99), the *mar1*Δ mutant (MAK1), and the *cap59*Δ mutant (cap59) strains were incubated in YPD medium at 30°C and CO_2_-independent medium (Gibco) at 37°C until saturation. Samples were fixed with 2.5% glutaraldehyde for 1 h at room temperature and were subsequently washed 3 times with PBS. Each sample was mounted onto 12-mm poly-l-lysine-coated coverslips (Neuvitro Corporation) and subsequently dehydrated by immersing the coverslips in ethanol (30% for 5 min, 50% for 5 min, 70% for 5 min, 95% for 10 min, 100% for 10 min, and 100% for 10 min). Samples were then critical point dried with a Tousimis 931 critical point dryer (Rockville, Maryland) and coated with gold-palladium using a Cressington 108 sputter-coater (Watford, United Kingdom). Coverslips containing the prepared samples were mounted and imaged on a Hitachi S-4700 scanning electron microscope (Tokyo, Japan).

### Cellular morphology defect quantification.

The WT (H99), the *mar1*Δ mutant (MAK1), and the *mar1*Δ + *MAR1* (MAK11) strains were incubated for 18 h in YPD medium at 30°C with shaking at 150 rpm. An OD_600_ of approximately 0.2 for each strain was transferred to 5 mL of fresh YPD medium and subsequently incubated at either 30°C or 37°C for 18 h with shaking at 150 rpm. Cells were then pelleted, washed with PBS, and imaged by DIC microscopy. DIC images were captured using a Zeiss Axio Imager A1 microscope equipped with an Axio‐Cam MRm digital camera. A minimum of 500 cells were analyzed across three biological replicates for each strain using the ImageJ software (Fiji). Statistical significance was determined using two-way analysis of variance (ANOVA) and the Tukey-Kramer test (GraphPad Software, San Diego, CA).

### Growth curve analysis.

The WT (H99), the *mar1*Δ mutant (MAK1), and the *mar1*Δ + *MAR1* (MAK11) strains were incubated for 18 h in YPD medium at 30°C with 150-rpm shaking. Cultures were normalized to an OD_600_ of 0.01 in 200 μL of fresh YPD medium and added to wells of a 96-well plate. Growth was then measured at an absorbance of 595 nm every 10 min for 40 h with shaking between readings and incubation at 37°C. Control wells containing YPD medium alone were also included to eliminate any background absorbance.

### Hypoxia resistance analyses.

The WT (H99), the *mar1*Δ mutant (MAK1), *mar1*Δ + *MAR1* (MAK11), and *sre1*Δ mutant (HEB6) strains were incubated in YPD medium at 30°C until the mid-logarithmic growth phase. Strains were washed once in PBS, normalized to an OD_600_ of 0.6 in 1 mL PBS, and serially diluted onto YES medium (0.5% [wt/vol] yeast extract, 2% glucose, and 225 μg/mL uracil, adenine, leucine, histidine, and lysine) agar plates with or without cobalt chloride (0.7 mM) (43). Microaerophilic conditions were generated using a sealed chamber (BD GasPak) and two activated GasPak EZ Campy container system sachets (43). Plates were placed in the chamber (microaerophilic) or outside the chamber (ambient air), incubated at 30°C, and imaged daily for 96 h. Cells incubated under ambient air conditions on YES medium and YES medium with cobalt chloride were imaged by DIC microscopy using a Zeiss Axio Imager A1 microscope equipped with an Axio‐Cam MRm digital camera. Cell diameter was measured using the ImageJ software (Fiji). A minimum of 50 cells were analyzed across two biological replicates for each fungal strain. Statistical significance was determined using two-way analysis of variance (ANOVA) and the Tukey-Kramer test (GraphPad Software, San Diego, CA).

### Ethical use of animals.

All animal experiments in this article were approved by the University of Texas at San Antonio Institutional Animal Care and Use Committee (IACUC) (protocol no. MU021), the Texas Christian University, the University of North Texas Health Sciences Center (UNTHSC) IACUC (protocol no. 1920-9), and the Duke University IACUC (protocol no. A102-20-05). Mice were handled according to IACUC guidelines.

### Data availability.

All fungal strains and reagents are available upon request.
