# Supplementary material for: An Immunogenic and Slow-Growing Cryptococcal Strain Induces a Chronic Granulomatous Infection in Murine Lungs
Source: Infect Immun. 2022 May 19;90(6):e00580-21. doi: 10.1128/iai.00580-21 (PMC9202370; doi:10.1128/iai.00580-21)
Supplement: Supplemental file 1 — supplemental material. Download iai.00580-21-s0001.pdf, PDF file, 5.9 MB [file iai.00580-21-s0001.pdf]

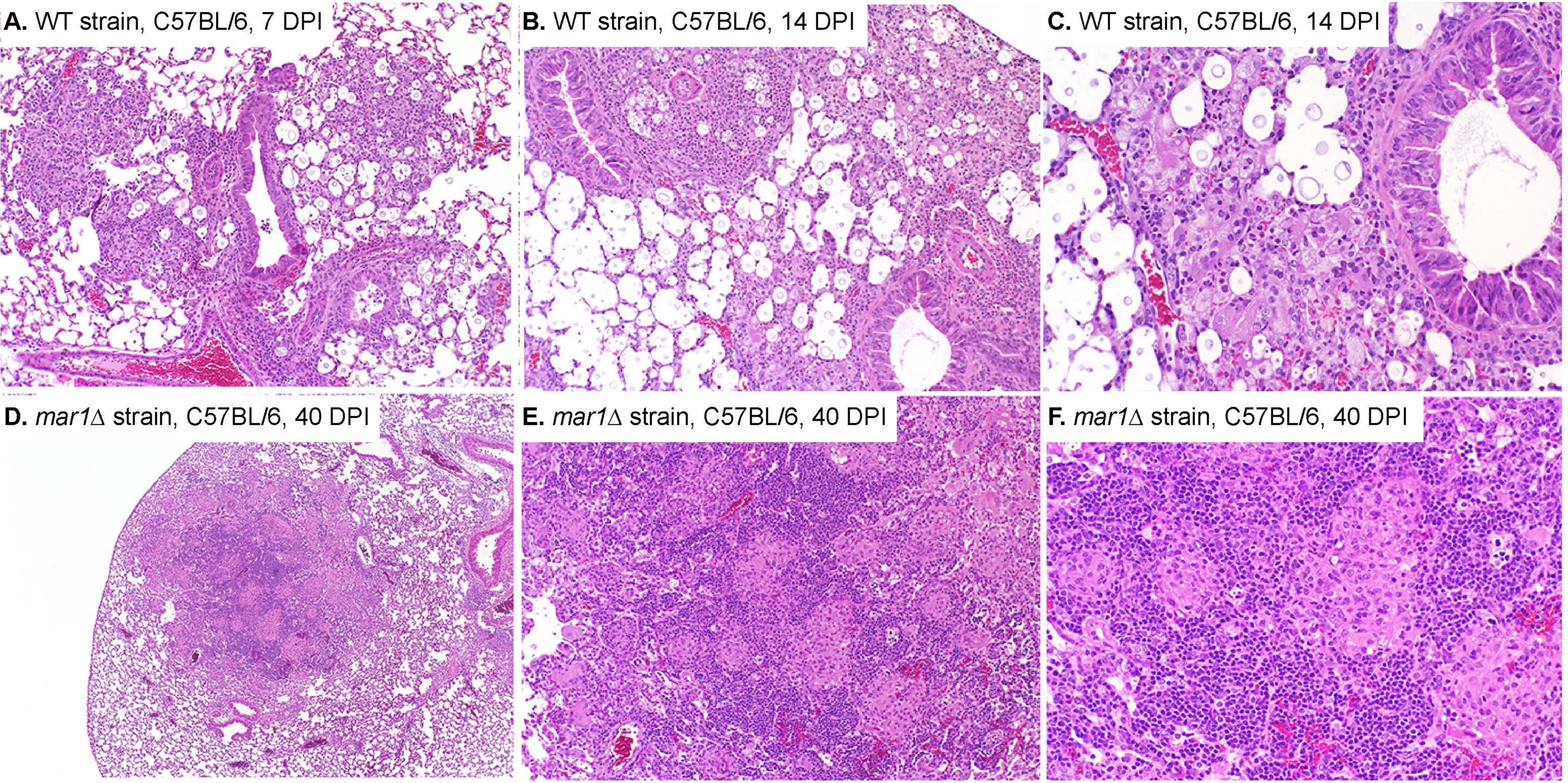

Figure S1. Additional hematoxylin- and eosin-stained histopathology images of the C57BL/6 granulomatous response. A. Lungs from a C57BL/6 mouse inoculated with 1 x 10^5 WT cells harvested at 7 DPI demonstrated a moderate peribronchiolar neutrophilic and mononuclear inflammatory reaction with vague, early, and poorly formed granulomatous region formation (10X). B. Lungs from a C57BL/6 mouse inoculated with 1 x 10^5 WT cells harvested at 14 DPI demonstrated moderate peribronchiolar nodular inflammatory aggregates accompanying a high organism burden (10X). C. A higher power image of (B) showed giant cells present without more discrete granuloma formation (20X). D. Lungs from a C57BL/6 mouse inoculated with 1 x 10^5 *mar1*Δ mutant cells harvested at 40 DPI showed a relatively well-circumscribed nodule containing well developed organizing lymphohistiocytic inflammation (4X). E. A medium power image of (D) highlighted compact histiocytic aggregates and peripheral mononuclear cells, characteristic of granuloma formation (10X). F. A higher power image of (E) further highlighted compact histiocytic aggregates and peripheral mononuclear cells, characteristic of granuloma formation (20X). DPI = days post-inoculation.

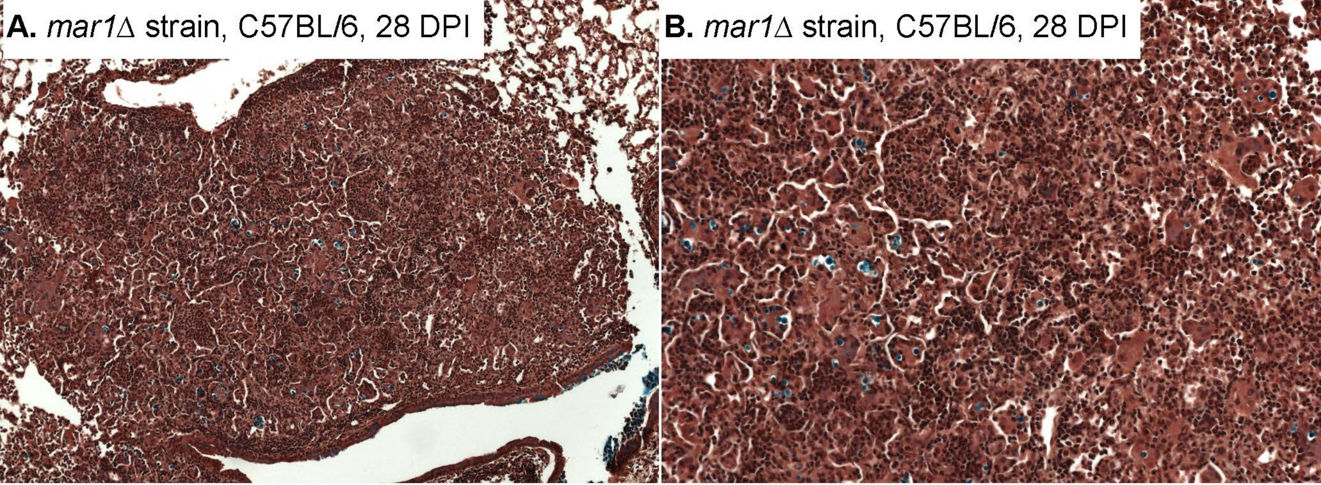

Figure S2. Movat-stained histopathology images of the C57BL/6 granulomatous response. A. Lungs from a C57BL/6 mouse inoculated 1 x 10^5 *mar1Δ* mutant cells harvested at 28 DPI demonstrated leukocyte recruitment (dark red), fibrotic tissue formation (light red), and fungal cell (blue) containment within the borders of the granulomatous region (10X). B. A higher power image of (A) (20X). DPI = days post-inoculation.

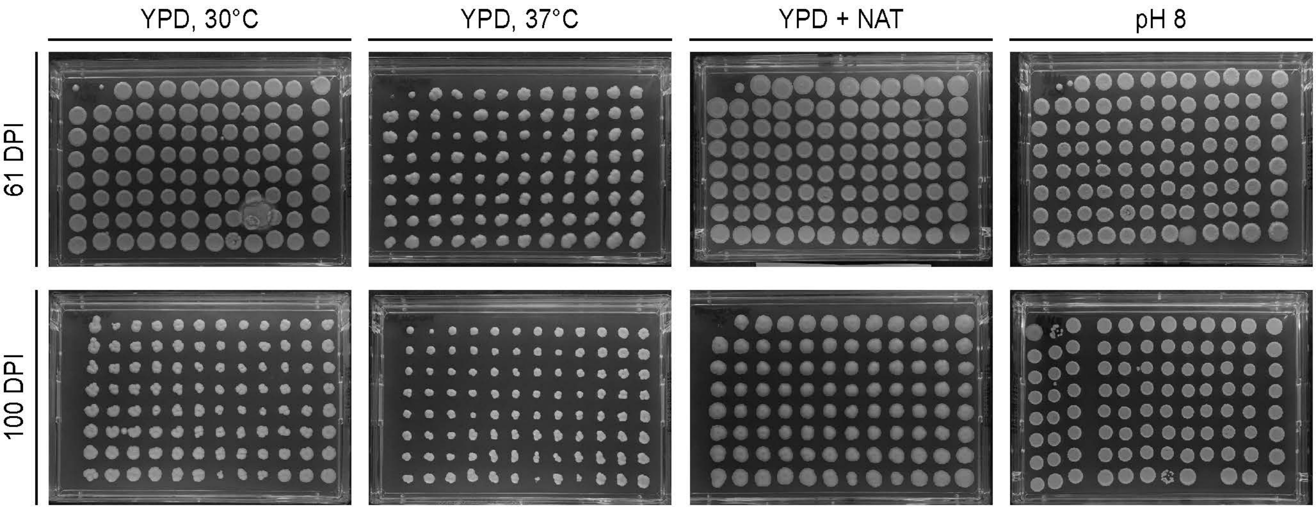

Figure S3. Recovery of *mar1*Δ mutant cells from murine lungs at extended timepoints in infection. Lungs from female C57BL/6 mice infected with 1 x 10^5 *mar1*Δ mutant cells were harvested at 61 and 100 DPI. Single fungal colonies were isolated on YPD agar plates and subsequently incubated in various conditions that allowed for identification of *mar1*Δ mutant isolates: YPD medium at 30°C, YPD medium at 37°C, YPD medium + nourseothricin (NAT), and YPD medium pH 8.15. The original WT strain (A1) and *mar1*Δ mutant strain (A2) are included as controls in each condition. DPI = days post-inoculation.

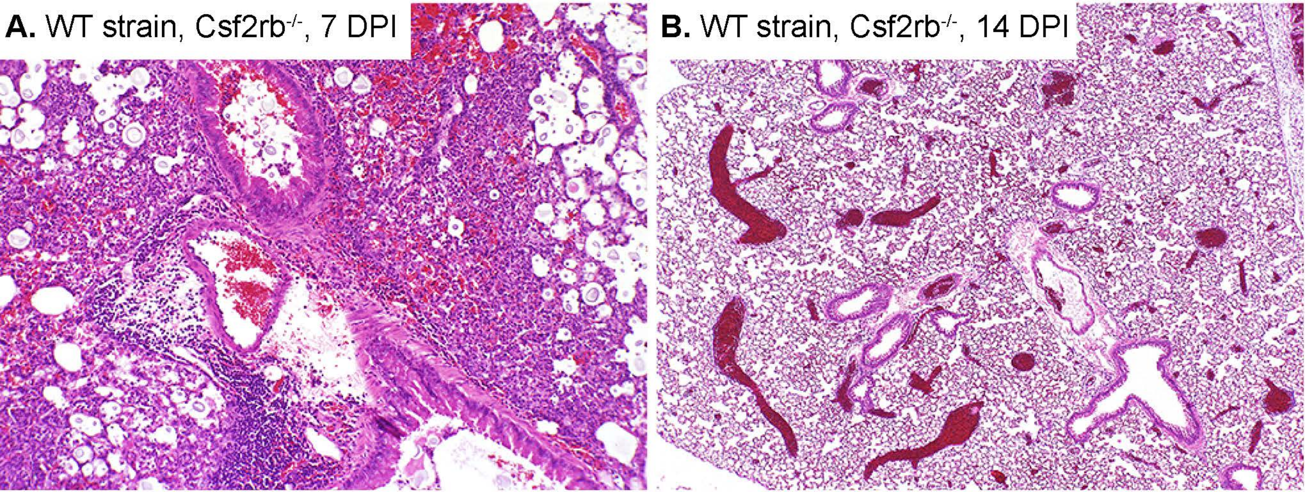

Figure S4. Additional histopathology images of the Csf2rb-/- granulomatous response. A. Lungs from a Csf2rb-/- mouse inoculated with 1 x 10^5 WT cells harvested at 7 DPI showed a marked peribronchiolar neutrophilic and mononuclear inflammatory reaction without granulomatous region formation (10X). B. Lungs from a Csf2rb-/- mouse inoculated with 1 x 10^5 WT cells harvested at 14 DPI demonstrated an absence of a significant inflammatory reaction (4X). All images are of hematoxylin- and eosin-stained tissue sections. DPI = days post-inoculation.

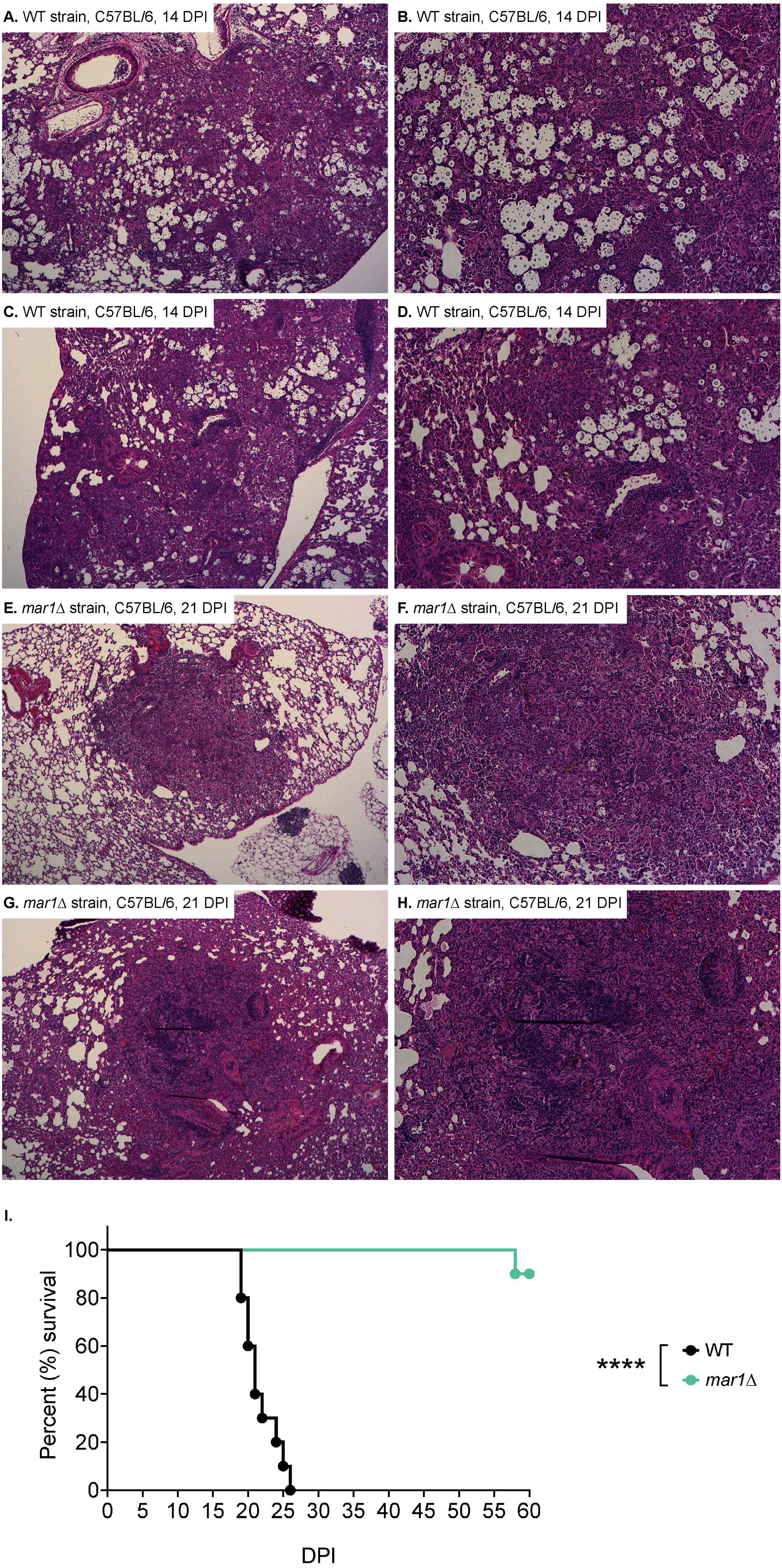

Figure S5. Murine infections with 1 x 10^4 CFU inoculum. A-D. Lungs from C57BL/6 mice inoculated with 1 x 10^4 WT cells harvested at 14 DPI demonstrated moderate inflammatory aggregates accompanying a high organism burden. Low power images (A & C) (4X) and medium power images (B & D) (10X) are included. E-H. Lungs from C57BL/6 mice inoculated with 1 x 10^4 *mar1*Δ mutant cells harvested at 21 DPI showed relatively well-circumscribed inflammatory nodules containing well developed organizing lymphohistiocytic inflammation. Low power images (E & G) (4X) and medium power images (F & H) (10X) are included. I. Female C57BL/6 mice (n = 10) were inoculated with 1 x 10^4 CFU of the WT strain or the *mar1*Δ mutant strain. Mice were monitored daily for 60 days and sacrificed at predetermined clinical endpoints predictive of mortality. Statistical significance was determined by log-rank test with Bonferroni correction (****, $P < 0.0001$). DPI = days post-inoculation.

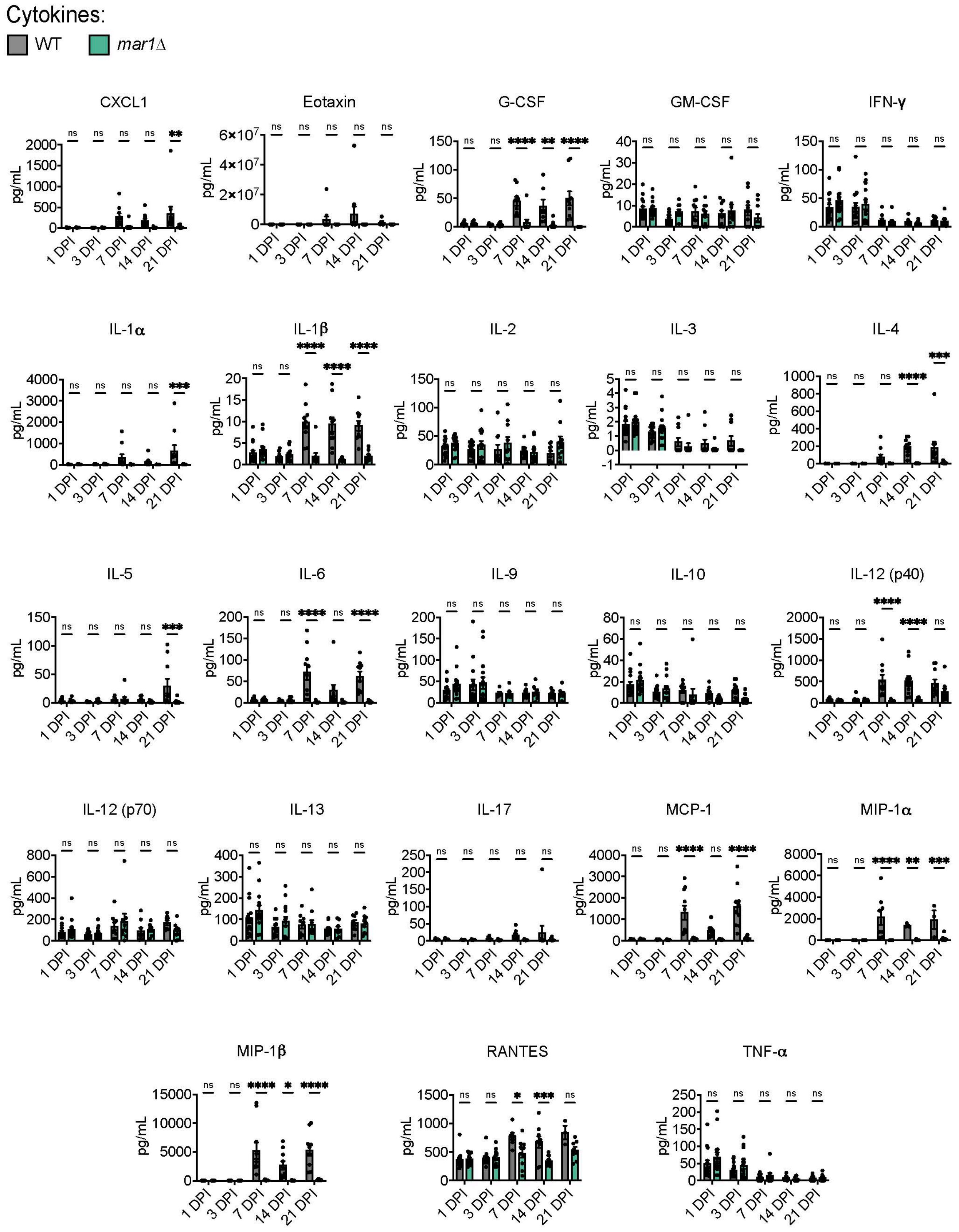

Figure S6. Complete pulmonary cytokine profile throughout infection. Pulmonary cytokine responses of female C57BL/6 mice inoculated with 1 x 10^4 cells of the WT strain or the *mar1*Δ mutant strain were measured using the Bio-Plex protein array system throughout infection: 1 (n = 15), 3 (n = 15), 7 (n = 10), 14 (n = 10), and 21 (n = 10) DPI. Error bars represent SEM. Statistical significance between strains at each timepoint was determined using two-way ANOVA (*, $P < 0.05$; **, $P < 0.01$; ***, $P < 0.001$; ****, $P < 0.0001$; ns, not significant). DPI = days post-inoculation.

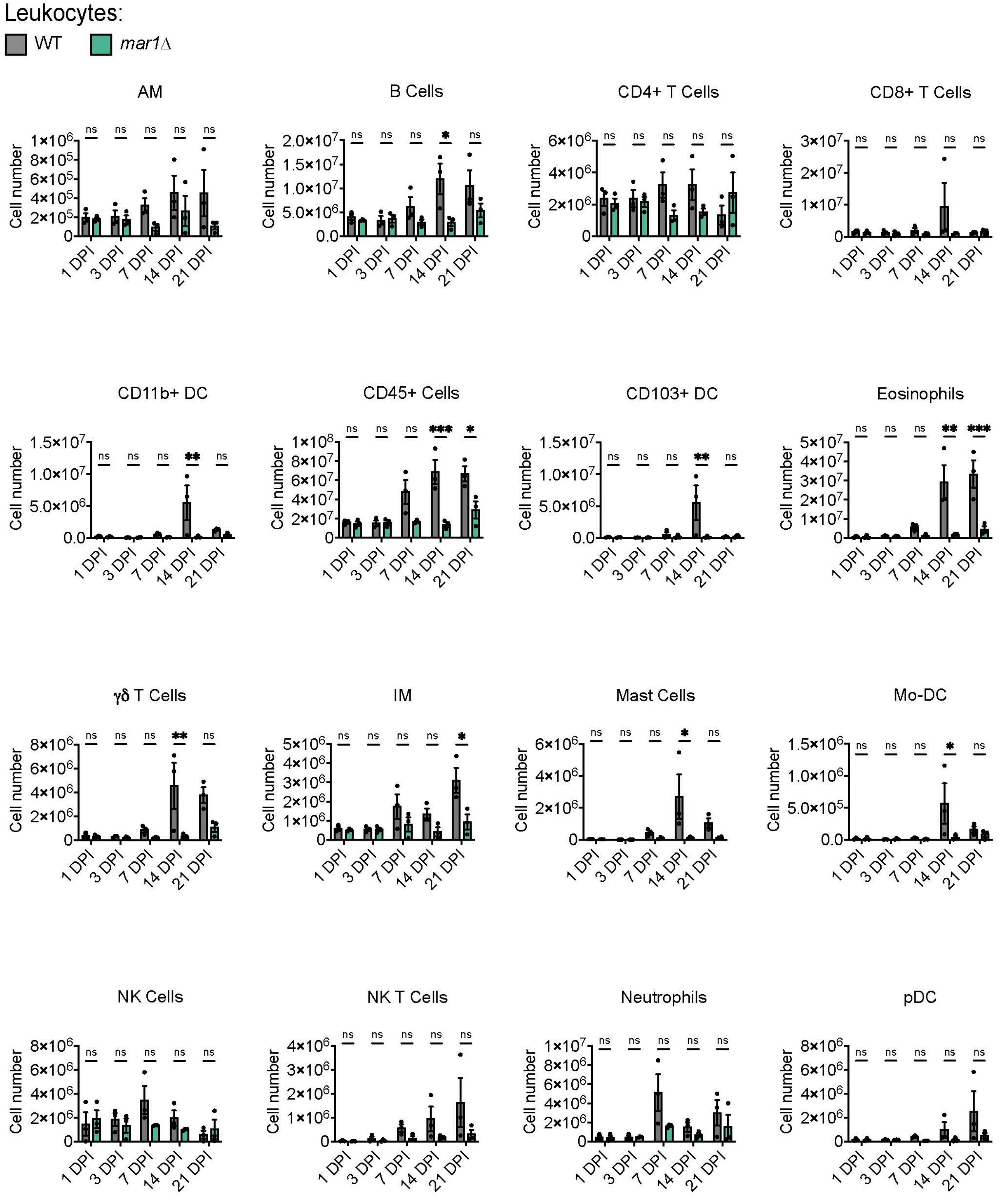

Figure S7. Complete pulmonary leukocyte infiltrate response throughout infection. Pulmonary leukocyte infiltrates of female C57BL/6 mice inoculated with 1 x 10^4 cells of the WT strain or the *mar1Δ* mutant strain were measured by flow cytometry throughout infection: 1, 3, 7, 14, and 21 DPI. Data shown are the mean ± of absolute cell numbers from three independent experiments (n = 3) performed using five mice per group per timepoint per experiment. Error bars represent the SEM. Statistical significance between strains at each timepoint was determined using two-way ANOVA (*, $P < 0.05$; **, $P < 0.01$; ***, $P < 0.001$; ns, not significant). DPI = days post-inoculation.

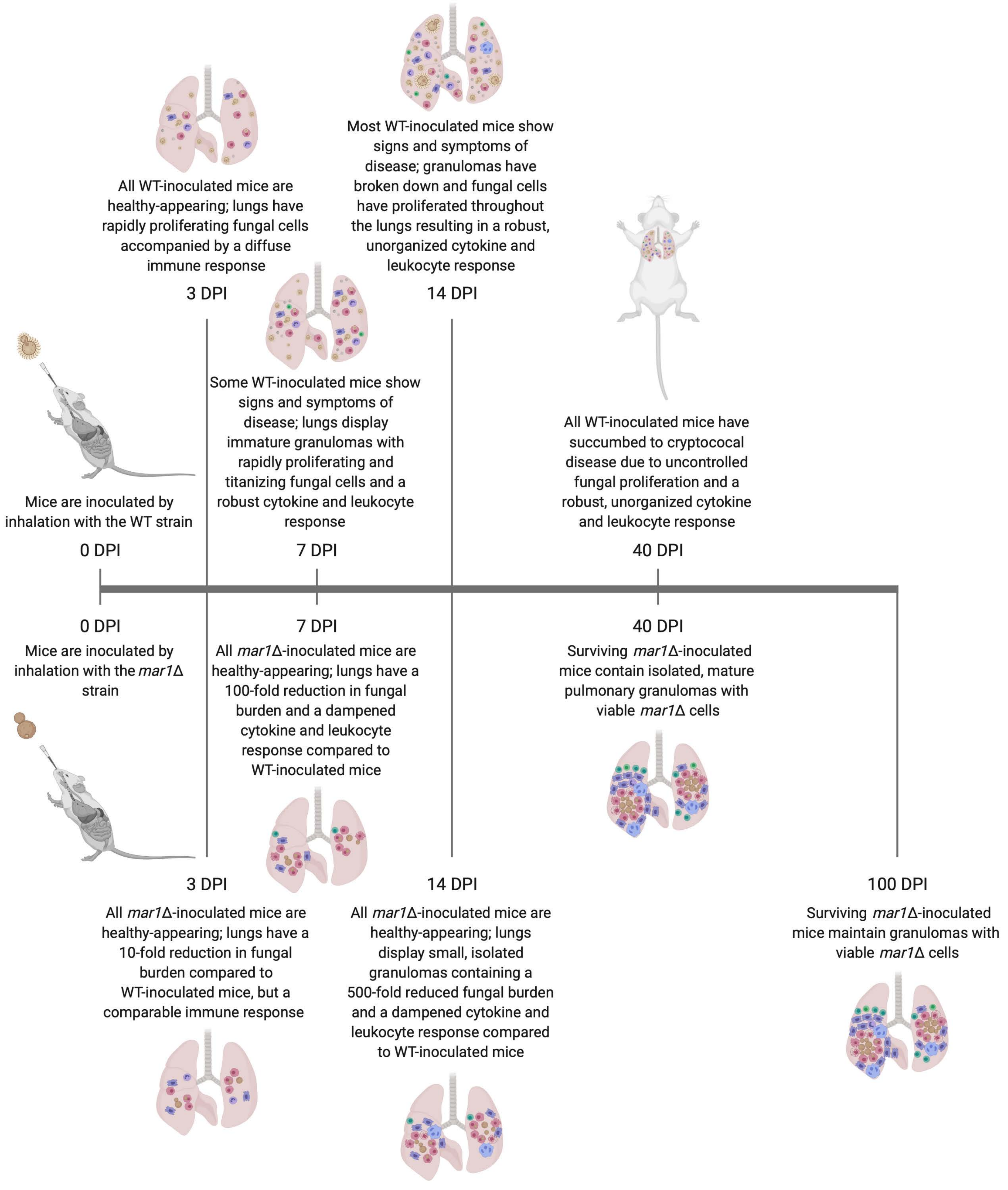

Figure S8. Granulomatous response timeline. Chronological summary of important observations about granulomatous region formation and maintenance in the WT strain (top) and *mar1*Δ mutant strain (bottom) backgrounds. Cartoons adapted from BioRender.com (2021). DPI = days post-inoculation.
